# Cep55 regulation of PI3K/Akt signaling is required for neocortical development and ciliogenesis

**DOI:** 10.1371/journal.pgen.1009334

**Published:** 2021-10-28

**Authors:** Behnam Rashidieh, Belal Shohayeb, Amanda Louise Bain, Patrick R. J. Fortuna, Debottam Sinha, Andrew Burgess, Richard Mills, Rachael C. Adams, J. Alejandro Lopez, Peter Blumbergs, John Finnie, Murugan Kalimutho, Michael Piper, James Edward Hudson, Dominic C. H. Ng, Kum Kum Khanna

**Affiliations:** 1 QIMR Berghofer Medical Research Institute, Herston, Australia; 2 School of Environment and Sciences, Griffith University, Nathan, Australia; 3 School of Biomedical Sciences, University of Queensland, St Lucia, Australia; 4 ANZAC Research Institute, Sydney, Australia; 5 Faculty of Medicine and Health, Concord Clinical School, University of Sydney, Sydney, Australia; 6 Discipline of Anatomy and Pathology, Adelaide Medical School, University of Adelaide, Adelaide, Australia; University of California San Diego, UNITED STATES

## Abstract

Homozygous nonsense mutations in CEP55 are associated with several congenital malformations that lead to perinatal lethality suggesting that it plays a critical role in regulation of embryonic development. CEP55 has previously been studied as a crucial regulator of cytokinesis, predominantly in transformed cells, and its dysregulation is linked to carcinogenesis. However, its molecular functions during embryonic development in mammals require further investigation. We have generated a *Cep55* knockout (*Cep55*^-/-^) mouse model which demonstrated preweaning lethality associated with a wide range of neural defects. Focusing our analysis on the neocortex, we show that *Cep55*^-/-^ embryos exhibited depleted neural stem/progenitor cells in the ventricular zone as a result of significantly increased cellular apoptosis. Mechanistically, we demonstrated that Cep55-loss downregulates the pGsk3β/β-Catenin/Myc axis in an Akt-dependent manner. The elevated apoptosis of neural stem/progenitors was recapitulated using Cep55-deficient human cerebral organoids and we could rescue the phenotype by inhibiting active Gsk3β. Additionally, we show that Cep55-loss leads to a significant reduction of ciliated cells, highlighting a novel role in regulating ciliogenesis. Collectively, our findings demonstrate a critical role of Cep55 during brain development and provide mechanistic insights that may have important implications for genetic syndromes associated with Cep55-loss.

## Author summary

Despite extensive investigation on the roles of CEP55 in tumorigenesis, its physiological role during development has remained largely uncharacterized. Recently, homozygous CEP55 mutations have been linked to two lethal fetal syndromes, MKS-like Syndrome and MARCH, demonstrating the importance of CEP55 in embryogenesis and neural development. These syndromes exhibit multiple severe clinical manifestations that lead to perinatal lethality. However, the exact molecular mechanism underlying complex Cep55-deficient developmental phenotypes remain elusive. To address this question, we have generated a Cep55^-/-^ (KO) mouse model and to bridge the gap between the mouse model and human disease, we have used brain organoids generated from pluripotent stem cells as a promising approach to investigate the mechanism of Cep55-associated neurodevelopment phenotype. Our detailed mechanistic studies suggest that Cep55 regulates neural development through the Akt-downstream effector, Gsk3β, and its mediators β-Catenin and Myc, which are known regulators of neural proliferation and differentiation. Additionally, we discovered a critical role for Cep55 in regulating cilia formation. Together, these results illustrate an important role of Cep55 in regulating neurogenesis and ciliogenesis via regulation of the Akt pathway.

## Introduction

Centrosomal protein 55 kDa (CEP55) is a crucial regulator of cytokinesis, the final stage of mitotic cell division [1]. CEP55 is highly upregulated in a wide spectrum of tumors and has been reported to play critical roles in the regulation of the *PI3K*/*AKT* pathway, stemness, genomic stability, and cell cycle progression [2,3]. In addition to the extensively investigated role of CEP55 in tumorigenesis, recent studies have examined developmental phenotypes resulting from CEP55 loss. Germline mutations of CEP55 in humans have been described in two lethal *CEP55*-associated syndromes, Meckel-Gruber syndrome (MKS)-like Syndrome [4,5] and MARCH (Multinucleated neurons, Anhydramnios, Renal dysplasia, cerebral hypoplasia, and Hydranencephaly) [6]. These syndromes exhibit severe clinical manifestations, including several congenital malformations, that lead to perinatal lethality. Homozygous nonsense mutations in *CEP55* that are predicted to lead to loss of protein were identified in affected fetuses. However, the mechanisms underlying CEP55-associated developmental phenotypes are not fully defined. We have generated a *Cep55*^-/-^ mouse model and cerebral organoids from pluripotent stem cells with or without depletion of CEP55 to investigate the mechanism of *CEP55*-associated neurodevelopment phenotype.

In mice and human cerebral organoids, we found that *Cep55* deletion resulted in a reduction in the size of mouse brains and human cerebral organoids due to excessive apoptosis of neural progenitor cells (NPCs). Additionally, we discovered a critical role for Cep55 in regulating cilia formation. Mechanistically, we show for the first time that Cep55 regulates neural development through the Akt-downstream effector, Gsk3β, and its mediators β-Catenin and Myc, which are known regulators of neural proliferation and differentiation [7]. In contrast, Cep55 regulation of ciliogenesis occurs through AKT independent of Gsk3β. Together, these results illustrate an important role of Cep55 in regulating neurogenesis and ciliogenesis in an Akt-dependent manner in mice.

## Results

### Loss of Cep55 leads to preweaning lethality in mice

**Fig 1:**
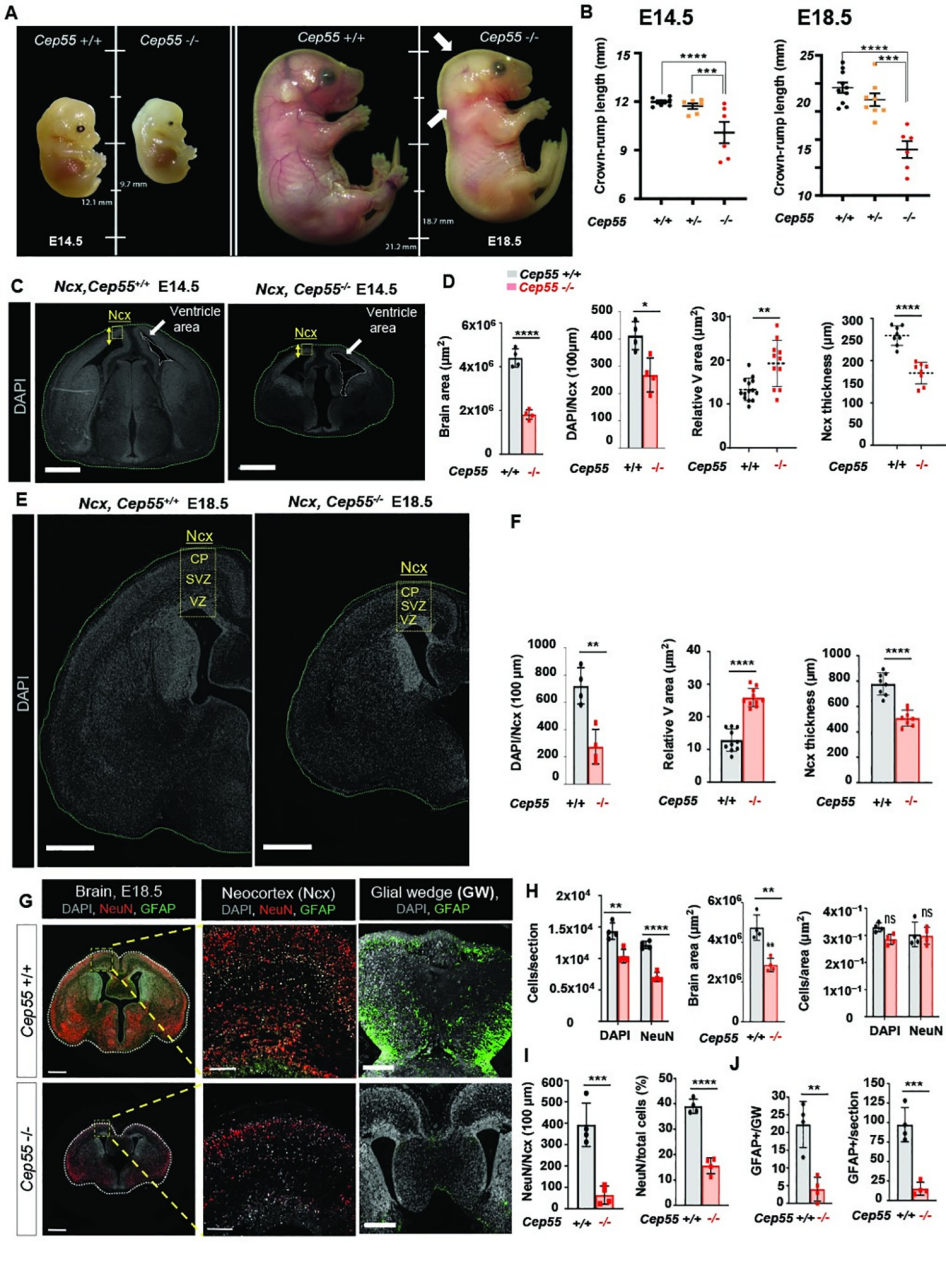
Loss of *Cep55* leads to preweaning lethality and microcephaly in mice. **(A)** Comparison of size (length, mm) and morphology of E14.5 (left) and E18.5 (right) *Cep55* ^+/+^ and *Cep55* ^-/-^ embryos. **(B)** Comparison of the crown-rump length (mm) of E14.5 (left, p< 0.0001) and E18.5 (right, p< 0.0001) *Cep55*^+/+^ and *Cep55* ^-/-^ embryos. Data represent the mean ± SD, n = 6–10 embryos per genotype. **(C,E)** Representative images of *Cep55*^+/+^ (left) and *Cep55*^-/-^ (right) neocortices (Ncx, yellow box) showing relative size of the Ncx. The yellow two-sided arrow represents the thickness of the Ncx, green dashed line shows the brain area, white dashed line shows the ventricle area and the yellow box resembles the analysis area, **(C)** Representative images of *Cep55*^+/+^ and *Cep55*^-/-^ mouse brains at E14.5, scale = 50μm. **(D)** Comparison of brain area of *Cep55*^+/+^ and *Cep55*^-/-^ E14.5 embryonic brains (left, p< 0.0001), and quantification of brain cell density (DAPI count within 100μm^2^ area of the neocortex) (Middle left, P< 0.0118) Quantification of the relative ventricle area (μm^2^; total area shown/total brain area) (Middle right, p< 0.0011), and Ncx thickness (right, p< 0.0001). **(E)** Representative images of *Cep55*^+/+^ and *Cep55*^-/-^ mouse brains at E18.5, scale = 700μm. **(F)** Comparison of *Cep55*^+/+^ and *Cep55*^-/-^ for left: total DAPI+ cell count in the Ncx, p< 0.0029; middle: relative ventricle area (μm^2^; total ventricular space/total brain area), p< 0.0001; and right: Ncx thickness, p< 0.0001. **(G)** Representative images of whole coronal section (left), boxed region at increased magnification (middle), and medial region/glial wedge (right) of E18.5 *Cep55*^+/+^ (upper) and *Cep55*^-/-^ (lower) embryonic mouse brains. Images show staining for NeuN (neuronal nuclei, mature neurons, red) and GFAP (Glial fibrillary acidic protein, marks astrocytes and ependymal cells, green). Right panel (glial wedge) shows the population of mature radial glia. Corpus callosum dysgenesis in Cep55^-/-^ brain (lower right), the boxed area in this panel shows GFAP expression in a magnified zone of glial wedge. Scale = 600 μm (left), Scale = 100 μm (middle), Scale = 400μm (right). **(H)** Comparison of *Cep55*^+/+^ and *Cep55*^-/-^ embryonic brain overall cell and neuron number per section (left, p< 0.0036), brain area (middle, p< 0.0020), and cell/neuron density (right, P> 0.02347 for DAPI and, P> 0.8632 for NeuN). N = 4 **(I)** Comparison of NeuN-positive neurons normalized to 100μm neocortical area (Ncx) in *Cep55*^+/+^ and *Cep55*^-/-^ E18.5 embryonic brains (left, p< 0.0010) and percentage of NeuN-positive cells across whole brain section normalized to the total number of cells identified by DAPI fluorescence (right, p< 0.0001). Data represent mean ± SD across two regions from n = 4 independent embryos per genotype. **(J)** Quantification of GFAP-positive cells in the glial wedge of *Cep55*^*+/+*^ and *Cep55*^*-/-*^ E18.5 embryonic brains (left, p< 0.0025) and GFAP-positive cells in the whole section (right, p< 0.0004). Data represent mean ± SD of four embryos, N = 4, average count of duplicate technical repeats, Student’s t-test, *p < 0.05, **p < 0.01, ***p < 0.001, ****p < 0.0001).

To investigate the physiological role of CEP55 during development, we generated a KO mouse model of *Cep55* using the “KO-first” allele design wherein the targeted allele acts as a gene-trap to form a non-functional allele (S1A Fig). Correct targeting was validated independently by genotyping PCR alongside the *Cep55* transcript and protein expression using RT-qPCR and immunoblotting analysis, respectively (S1B, S1C and S1D Fig). To generate the colony of *Cep55*^*-/-*^ mice, we intercrossed *Cep55*^*+/-*^ mice, with the expectation that approximately 25% of the offspring would be of a *Cep55*^-/-^ genotype according to Mendelian ratios. Interestingly, after genotyping more than 321 offspring from these breedings (postweaning), we did not obtain any viable *Cep55*^-/-^ mice, indicating that genetic loss of *Cep55* led to embryonic or preweaning lethality (S1 Table). To define the time point of lethality, pregnant dams from *Cep55*^+/-^ intercrosses were euthanized at different stages of pregnancy, ranging from E11.5—E18.5, and embryos collected for phenotypic evaluation. Interestingly, we were able to obtain viable *Cep55*^-/-^ offspring at each gestational stage (E11.5 -E18.5) (S2 Table). Embryos collected at both E14.5 and E18.5 exhibited significant dwarfism, based on crown-rump length measurements, when compared to control (*Cep55*^*+/+*^) embryos. Additionally, *Cep55*^-/-^ embryos exhibited an increased thickness of the neck and a flattened head (Fig 1A and 1B)). Notably, we found 1 KO pup (3.4%) out of 29 pups at P0; hence, we observed only a rare incidence of homozygous pups born. In previously published studies, homozygous Cep55^-/-^ mice were born at Mendelian ratio, although differences in timing and penetrance of postnatal lethality have also been reported [8–10], which will be further elaborated in the discussion section.

To determine if the loss of a single allele of *Cep55* would cause phenotypic changes, we performed the histological examination of multiple organs from eight-week-old *Cep55*^+/-^ mice relative to *Cep55*^+/+^ littermates. We observed no significant differences in the pathohistology or size of respective organs (S1E Fig) indicating that loss of a single allele of *Cep55* does not impact physiological development. Additionally, the monitoring of both genotypes showed no significant differences in body weight for the first 20 weeks (S1F Fig). These data suggest that a single allele of *Cep55* is largely sufficient to maintain physiological functions.

Several recent reports have shown that CEP55 functional loss in humans leads to a range of congenital abnormalities, all with defective brain development [4–6]. Therefore, we next sought to examine the expression pattern of *Cep55* using single-cell transcriptomic data of mouse neocortical development [11]. This analysis revealed that *Cep55* expression levels are highest in the NPCs of E14 embryos (S1G Fig). Moreover, investigating human fetal brain data from Allen Brain Atlas revealed that expression of *Cep55* peaks from weeks 8–10 of gestation, followed by a reduction after 16 weeks. Minimal detection was reported between weeks 27–35, but expression became detectable again three weeks prior to birth [12]. This expression pattern corresponds with the timing of human neurogenesis in the neocortex through neurogenic divisions and neuronal differentiation from radial glial cells (RGCs) [13]. To validate the expression of *Cep55* during development, we performed β-galactosidase staining in the *Cep55*^+/-^ mice, as the targeted allele contains a LacZ reporter. In the isolated brain of mouse embryos, a gradient of expression of *Cep55* in the neocortex was detected at E12.5, diminishing at E14.5 to become undetectable at E16.5 (S1H Fig). Collectively, our data suggest that Cep55 plays a critical role during neurogenesis.

### *Cep55* loss causes gross morphological defects in mouse embryos

Given the neurodevelopmental expression pattern of *Cep55*, we predicted that significant neural deficits would arise from Cep55 loss. To investigate this, we first performed hematoxylin and eosin (H&E) staining of sagittal and coronal sections from E18.5 *Cep55*^*+/+*^, *Cep55*^*+/-*^ and *Cep55*^*-/-*^ mice. We observed no gross morphological differences in the lung, intestine or liver among the respective genotypes. However, we noted prominent abnormalities in the brain of *Cep55*^-/-^ embryos when compared to respective controls, which were characterized by a partial failure (hypoplasia) and disorder (dysplasia) of normal structural brain development. The cerebellum was hypoplastic, with marked thinning of the germinative external-granular layer (EGL), the secondary germinal zone that produces granule cell progenitors, and a diminution and disorganization of neurons (S2A Fig, right). In addition, the neuronal population of the olfactory bulb was disorganized and depleted (S2A Fig, left). Furthermore, coronal sectioning of the brain revealed neocortical depletion of neurons and ventricular dilatation, as well as smaller germinal regions in both dorsal and ventral telencephalon (S2B Fig). The neocortices of *Cep55*^-/-^ brains were hypoplastic and dysplastic, with diminished and disorganized neurons. In addition to apoptosis in the neocortex, there were also multifocal areas of cortical necrosis and parenchymal loss, with evidence of phagocytosis of affected neurons by macrophage-like cells (S2C Fig, upper). Numerous bi-nucleated neurons were also evident in the neocortex of *Cep55*^-/-^ mice (S2C Fig, lower). To measure this defect, we stained mature neurons with NeuN (RBFOX3) and quantified the number of bi-nucleated neurons in the neocortex of brain.The proportion of multinucleated neurons expressing NeuN in the cortical region of *Cep55*^*-/-*^ mice was increased compared to that of *Cep55*^+/+^ (S2D and S2E Fig), a phenotype reminiscent of the described changes in human embryos with MARCH syndrome [6].

As cortical neurogenesis peaks at approximately E14.5 [14,15] and the highest expression of *Cep55* was found at this embryonic stage (S1G Fig), we chose this gestational stage for characterizing the phenotypes and cellular behaviors in Cep55-deficient mice. Also, to better characterize the specific disruption to neural cells, we focused our investigation on the neocortex, a well-characterized region of the developing forebrain with prominent *Cep55* expression. Strikingly, brain sizes of *Cep55*^-/-^ E14.5 embryos were found to be significantly smaller compared to that of *Cep55*^+/+^ by measuring the brain area (Fig 1C and 1D). We also found a reduced number of cells in the neocortex of *Cep55*^-/-^ embryos. Furthermore, the size of the ventricle relative to the total brain area was larger and dilated in *Cep55*^-/-^ mice, consistent with our previous histopathological observations. We also observed that the thickness of the cortex was reduced in *Cep55*^-/-^ brains compared to *Cep55* ^+/+^ (Fig 1C and 1D). Similar to our observations at E14.5, we noted fewer cells (DAPI) in the cortex of *Cep55*^-/-^ embryos compared to *Cep55* ^+/+^ at E18.5 (Fig 1E and 1F). Consistently, the ventricles were larger and dilated in *Cep55*^-/-^ brains and cortex thickness was reduced (Fig 1E and 1F). Also, brain sizes of *Cep55*^-/-^ embryos at E18.5 were found to be significantly smaller compared to that of *Cep55*^+/+^ (Fig 1G and 1H). Interestingly, this size reduction in *Cep55*^-/-^ brain area likely resulted from decreases in the number of both total cells (DAPI stained) and neurons (NeuN stained), since the density of the cells (cells/area) after normalization to total brain area was not significantly different between genotypes (Fig 1G and 1H).

To further investigate the reduction of cell populations, we stained brain sections for NeuN to mark mature neurons and glial fibrillary acidic protein (GFAP) to mark astrocytes. The mature neurons (NeuN-positive cells) were reduced in numbers in the neocortex in *Cep55*^*-/-*^ compared to that of *Cep55*^+/+^ embryos, even after normalization to the total cell number as assessed by DAPI staining (Fig 1G and 1I). Interestingly, GFAP-positive cells in the neocortex were reduced in *Cep55*^-/-^ embryos when compared to *Cep55*^+/+^ (Fig 1G and 1J), suggesting broader defects in the development of the central nervous system. In the cortical region, GFAP can be a marker of either astrocytes or the radial-glial-like neuronal stem cells and represents mature radial glia which can be seen in the medial region (e.g. glial wedge). We also observed a drastic reduction in GFAP-expressing cells at the cortical midline and in the neocortex in *Cep55*^*-/-*^ brains compared to that of *Cep55*^+/+^. These cells are critical in facilitating the crossing of axons through the corpus callosum [16]. In line with the lack of GFAP-expressing cells, at the midline, we observed dysgenesis of the corpus callosum in mutant mice at this age (Fig 1G). Taken together, these data show that loss of Cep55 results in defective neuropathological phenotypes in mice.

### Cep55 regulates the fate of radial glial and intermediate progenitor cells

**Fig 2:**
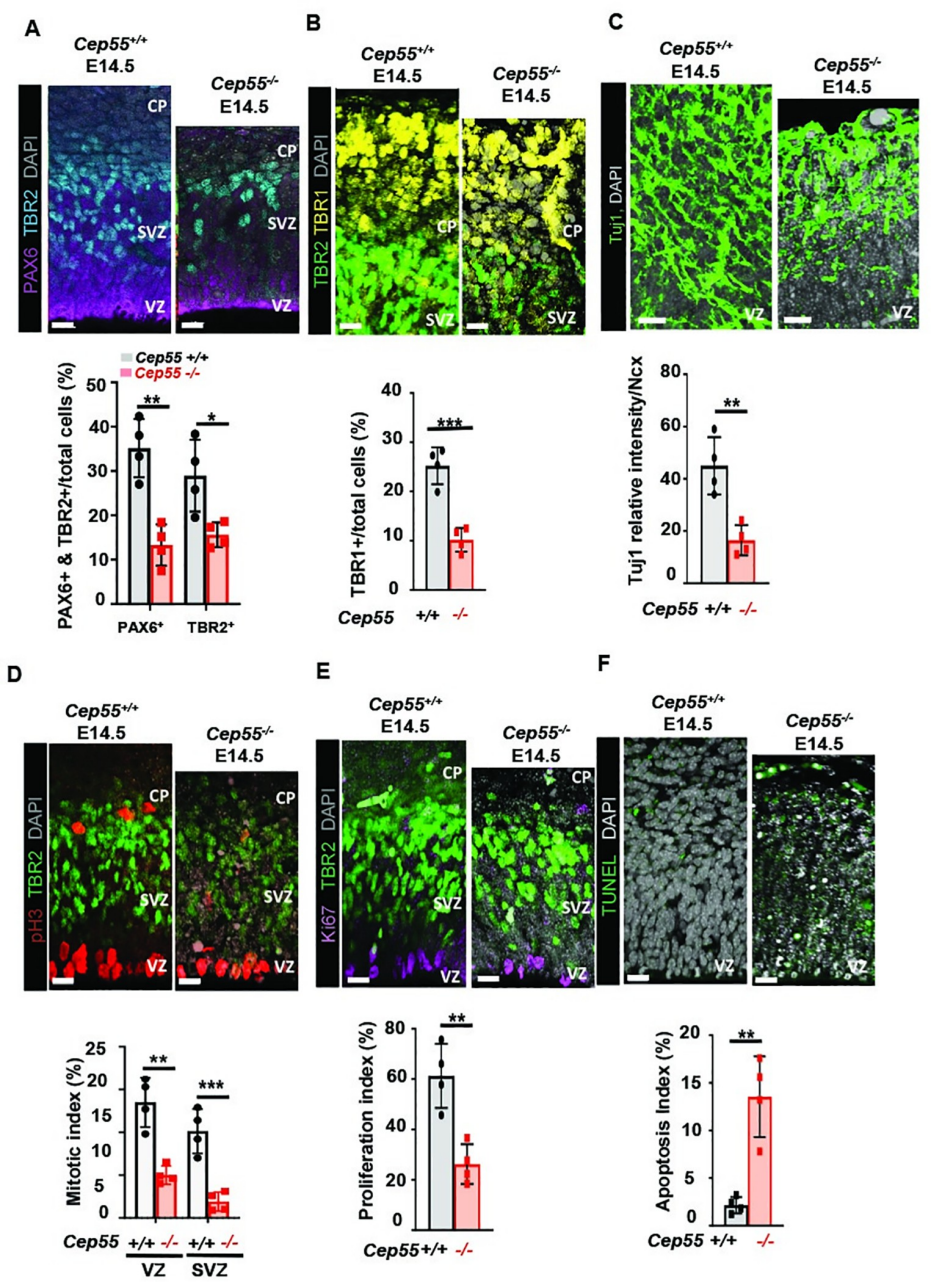
*Cep55* regulates cell fate of radial glial and intermediate progenitor cells, and neurons. **(A)** Representative images of radial glial cells (RGC; Pax6+) at VZ, intermediate progenitor cells (IPC; Tbr2+) at SVZ and total cells (DAPI) at E14.5, scale = 15 μm (upper panel). Quantification of percentage of RGCs (Pax6+, p< 0.0016) and IPCs (Tbr2+, p< 0.0337) in *Cep55*^+/+^ and *Cep55*^-/-^ neocortices (Ncx) at E14.5 (lower). **(B)** Representative images of IPCs (Tbr2+) at SVZ, post-mitotic neurons (Tbr1+) at CP and total cells (DAPI, gray) at E14.5, scale = 15 μm (upper), quantification of the percentage of post-mitotic-neurons (Tbr1+, p< 0.0005) in *Cep55*^+/+^ and *Cep55*^-/-^ neocortices at E14.5 (lower). **(C)** Neuron-specific class III β-tubulin (Tuj1, p< 0.0037), relative intensity of Tuj1 staining quantified within a 100 μm^2^ field of view. **(D)** Phosphohistone H3 (pH3; mitotic cells) in the VZ and SVZ, co-stained with Tbr2 to identify proliferating IPCs at E14.5, scale = 15 μm. Quantification of the percentage of total cells expressing pH3 to show the mitotic index in Vz, p< 0.0090; and SVZ, P< 0.0070. **(E)** Proliferating cells (Ki67+), IPCs (Tbr2+, green) delineating the SVZ, and total cells (DAPI) at E14.5, scale = 15 μm (upper), Comparison of the proportion of Ki67+ cells in *Cep55*^+/+^ and *Cep55*^-/-^ neocortices to show proliferation index (lower, p< 0.0034). **(F)** Apoptotic cells (TUNEL) and total cells (DAPI) in the Ncx, at E14.5, scale = 15 μm (upper), comparison of proportion of apoptotic cells in *Cep55*^+/+^ and *Cep55*^-/-^ neocortices to show apoptosis index (lower, p< 0.0019).

Having observed reduced numbers of neurons and astrocytes in the cortex of *Cep55* mutant mice, we next sought to determine how Cep55 regulates NPCs differentiation and development during neurogenesis, with a focus on the different neuroepithelial layers of the neocortex during embryonic development. Neurogenesis in the developing neocortex occurs with the contribution of two types of NPCs: radial glial cells (RGCs) and intermediate progenitor cells (IPCs) [17]. The former divide at the ventricular zone (VZ; the apical surface), and express the homeodomain transcription factor, Pax6. The latter, which are derived from RGC and only produce neurons, divide within the basally located subventricular zone (SVZ) and express Tbr2, a T-domain transcription factor. The subsequent transition from IPCs to postmitotic projection neurons (PMN) in the cortical plate (CP) is marked by the onset of Tbr1 expression [17]. We investigated the different populations of progenitor cells within the nascent cortex, to determine how a deficit in cortical neuron number might arise in our mutants. We categorized RGCs as the Pax6^+^ Tbr2^–^ population, as some newborn Tbr2^+^ IPCs retain transient expression of Pax6 [18]. Immunostaining for Pax6, Tbr2, and Tbr1 at E14.5 revealed a reduction in the number of RGCs, IPCs, and post-mitotic neurons as a proportion of the total cortical cell number in *Cep55*^-/-^ mice compared to Cep55^+/+^ controls (Fig 2A and 2B). Furthermore, we also observed a reduced population of neurons marked by Tuj-1, the neuron-specific class III β-tubulin, in *Cep55*^-/-^ neocortices compare to *Cep55*^+/+^ (Fig 2C). Next, we investigated how *Cep55* loss impacts mitosis, proliferation, and apoptosis of NPCs by staining for phosphohistone H3-S10 (pH3), Ki67, and TUNEL, as markers of these cellular processes respectively. We found a reduced proportion of pH3 positive-cells in *Cep55*^-/-^ mice, when the pH3-positive cell pool was normalized to the total number of cells. This is indicative of either mitotic defects or delayed mitosis/cytokinesis in the absence of Cep55 (Fig 2D). Consistently, the proliferation index at E14.5 was significantly reduced in *Cep55*^-/-^ animals (Fig 2E). Finally, we found high levels of apoptosis as marked by TUNEL staining in *Cep55* ^-/-^ neocortices compared to controls at E14.5 when assessed as a percentage of total cell number (Fig 2F). Interestingly, western blot analysis of mouse embryonic brain (E14.5) extracts also showed upregulation of cleaved caspase-3 in *Cep55*^-/-^ brains consistent with our IHC results and histopathological observations (S2F Fig). Collectively, these data suggest that reductions in NPCs are due to elevated levels of cell death and reduced proliferation and indicate a role for Cep55 in the survival and viability of neural progenitor populations during neocortical development.

### Elevated apoptosis following *CEP55* knockdown in human cortical organoids

**Fig 3:**
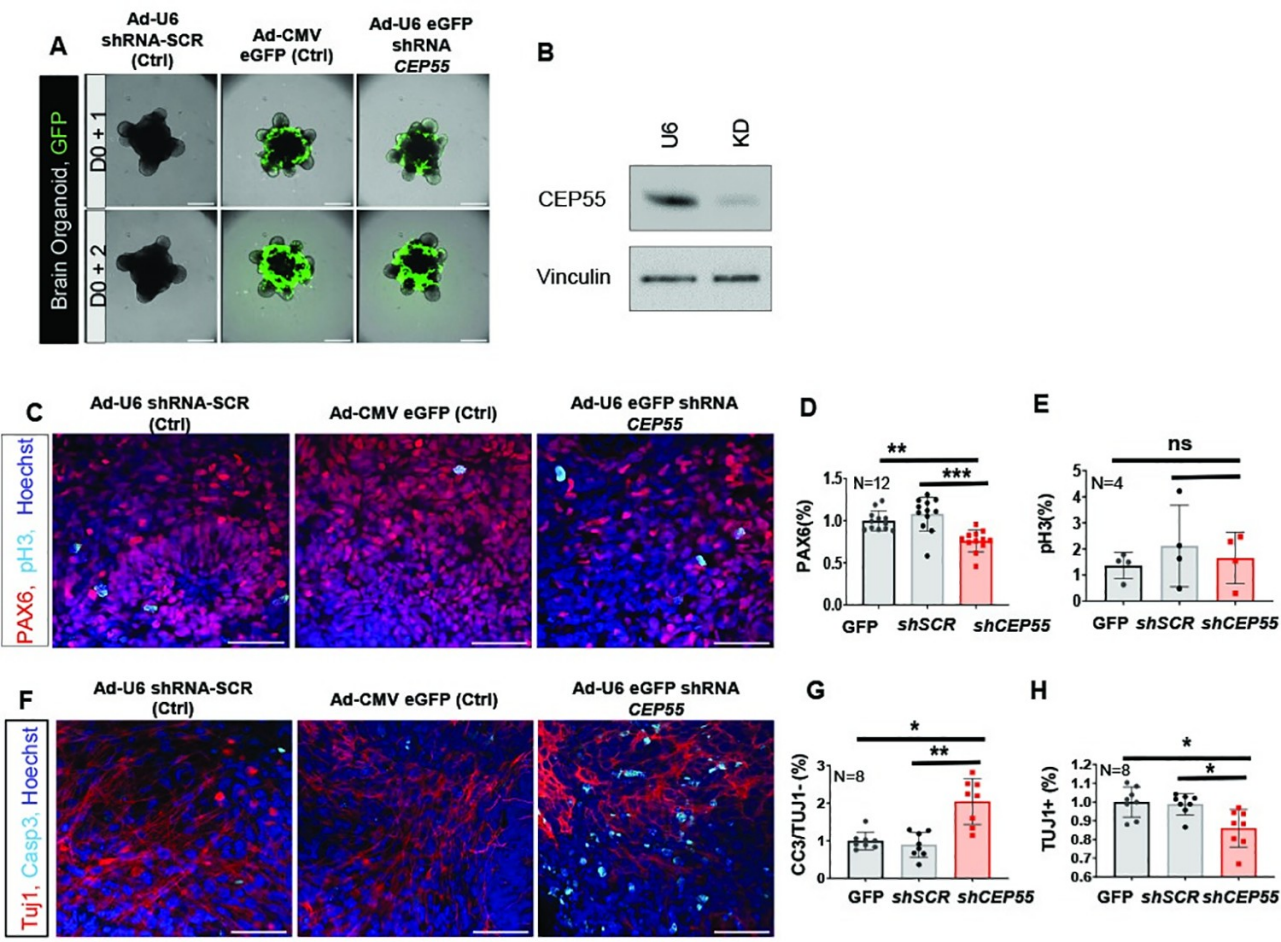
*CEP55* knockdown induces cell death in neural progenitors of human cerebral organoid. **(A)** Day 16 of human iPSC-derived cerebral brain organoids infected with U6 scrambled control (left), CMV GFP control (middle) and CEP55 knockdown (KD) (right) adenoviral shRNAs with GFP-tag. Expression levels of integrated viral GFP increased significantly between days 1 to 2 post-infection at a MOI of 10. shRNA U6 scrambled control did not contain a GFP tag. Upper panel: D0 (infection day) +1; lower panel: D0 +2, scale bars = 50μm, immunofluorescent labeling of cerebral organoids was done after 24 hours of shRNA infection. The brain organoid differentiations were repeated as 3 entirely separate differentiations, each containing 192 technical replicates towards all the described experiments. Each technical organoid replicate was also cultured independently for the described 18 days post induction. **(B)** Immunoblot shows knockdown of CEP55 in adenoviral shRNA against CEP55 transduced organoid compare to U6 scrambled control. Vinculin serves as the loading control. **(C)** Representative images of labeling for neural progenitor PAX6, mitotic marker pH3 and nuclear Hoechst showed a decrease in neural progenitor PAX6 in the CEP55 KD shRNA infected organoids compared to the U6 and GFP controls. **(D-E)** Quantified immunolabelled organoids (normalized to CMV GFP control) for **(D)** PAX6, p< 0.0045 (GFP) and, P< 0.0001 (shSCR) and **(E)** pHH3, p> 0.9804 (GFP) and, P> 0.999 (shSCR). **(F)** Representative images of labeling for neural-specific tubulin TUJ1, apoptotic marker cleaved caspase 3, and nuclear Hoechst showed a clear increase in cell death in the CEP55 KD samples compared to the controls. **(G-H)** Quantified immunolabelled organoids (normalized to CMV GFP control) for **(G)** Cleaved Caspase-3 in non Tuj1+ cells, p< 0.0100 (GFP) and P< 0.0027(shSCR); TUJ1/Caspase3 double positive cells by first identifying a TUJ1+ cell by the presence of a positively stained nucleus (Hoechst staining) and subsequently counting whether they were double positive for Caspase3, **(H)** Total Tuj1+ cells, p< 0.0157 (GFP) and p< 0.0362 (shSCR). For all calculations, data represented mean ± SD, the number of organoids indicated in figures. One-way ANOVA (Kruskal–Wallis test) performed; *p< 0.05, ** p < 0.01, scale = 50 μm.

We next investigated the effect of *CEP55*-loss in human cerebral brain organoids generated from embryonic pluripotent stem cells (HES3). Cerebral organoids mimic the unique and dynamic features of early human cortical development in culture, enabling detailed analysis of organ pathogenesis due to particular genetic deregulation or dysfunction [19]. We performed knock-down of *CEP55* in differentiated HES3 organoid cultures using adenoviral GFP-tagged shRNA against *CEP55* or a scrambled control (U6). Knockdown was performed after cerebral organoid induction to circumvent potential early apoptosis caused by *CEP55* loss. Within 24–48 hours of adenoviral transduction, we observed a significantly increased level of the integrated virus as marked by GFP expression for control CMV-eGFP and sh-*CEP55* (shRNA U6 scrambled control did not contain a GFP tag) (Fig 3A). The enlarged images illustrate the neuroepithelial structures (S2G Fig) and differentiation in brain organoids using specific markers to detect progenitor cells (PAX6, TBR2) and differentiated neurons (TBR1 and chicken ovalbumin upstream promoter transcription factor-interacting proteins 1; CTIP-1) (S2H Fig). Given the extent of apoptosis observed in our mouse model, we characterized the effect of *CEP55* knock-down (Fig 3B) in the cerebral organoids at 24 hours post-infection. A significant reduction of PAX6-positive NPCs was observed within 24 hours of *CEP55* shRNA transduction in organoids compared to control (Fig 3C and 3D). However, we did not find a significant difference in pH3 positive cells (Fig 3C and 3E). The reduced number of PAX6 cells in organoids transduced with *CEP55* shRNA was likely caused by a dramatic increase in cell death (cleaved caspase 3) (Fig 3F and 3G). Consequently, we observed a significant decrease in TUJ1+ neurons in *CEP55*-knockdown organoids compared to controls (Fig 3F and 3H). Collectively, our findings of reduced RGC numbers due to apoptotic cell death in human cerebral organoids were consistent with the *Cep55*^*-/-*^ phenotype observed in mice.

### Cep55^-/-^ mice exhibit cilial abnormalities

**Fig 4:**
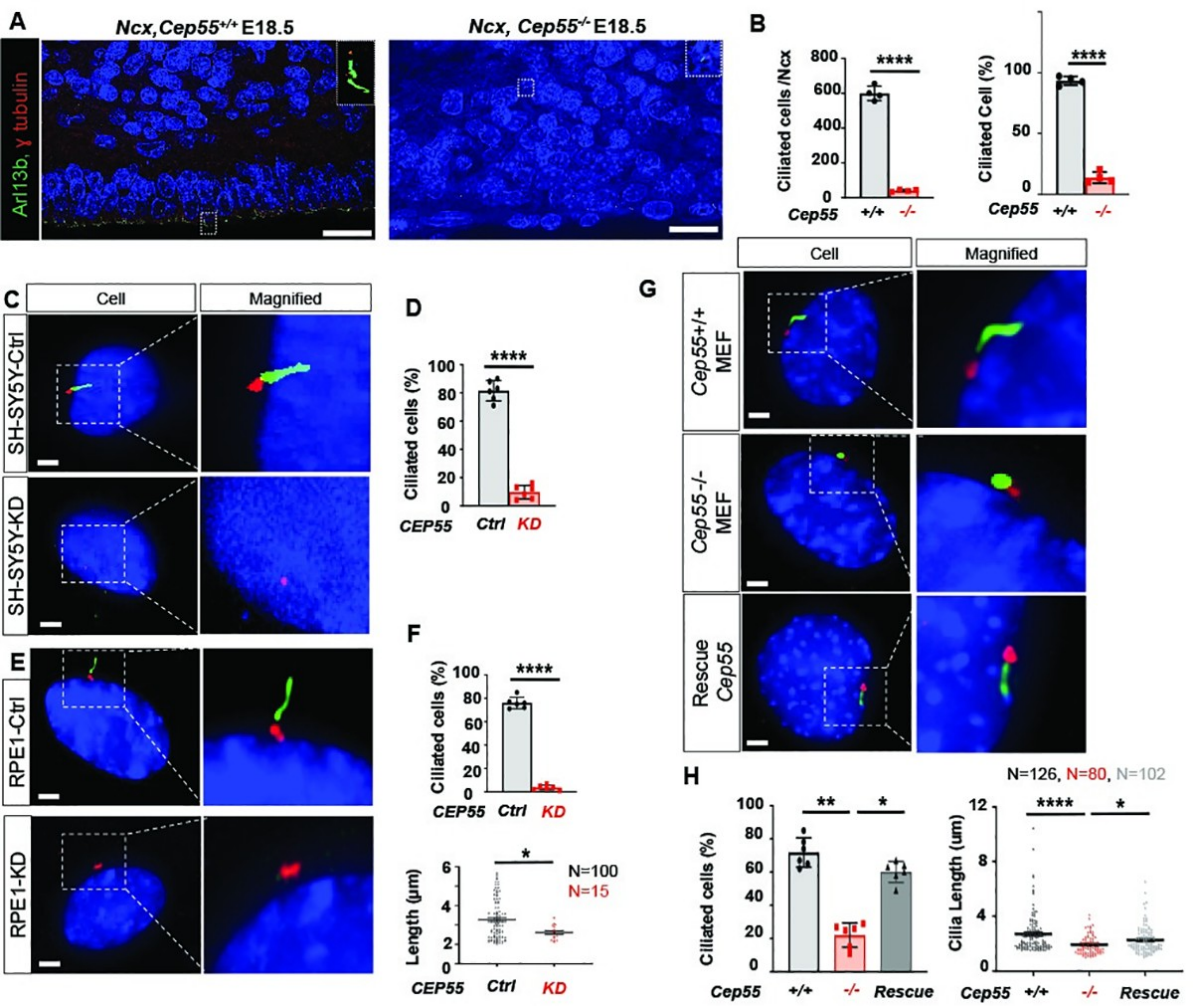
*Cep55* is localized to basal body of cilia and regulates ciliogenesis. **(A)** Super-resolution microscopy of *Cep55*^*+/+*^ and *Cep55*^*-/-*^ mouse neocortex at E18.5 immunostained for cilia (Arl13b), basal body (Ɣ-tubulin), and DAPI, scale = 10 μm. **(B)** Percent of cilia-positive cells in Ncx at E18.5, p< 0.0001. **(C-H)** Representative images showing cilia (Arl13b), basal body (Ɣ-tubulin), *Cep55* (yellow), and DAPI (blue), Scale = 5 μm. **(C)** Representative image of cilia and basal body in control (upper) and *CEP55* knockdown (lower) in SH-SY5Y cells. **(D)** Percentage of ciliated cells in Ctrl and *CEP55* knockdown SH-SY5Y, p< 0.0001. **(E)** Cilia, basal body, and CEP55 staining in RPE-1 cells transfected with a control lentiviral vector (Ctrl) (upper), RPE-1 cells with lentiviral knockdown of *CEP55* (lower). **(F)** Percentage of ciliated RPE-1 cells with or without *CEP55* knockdown (upper, P< 0.0001), Scatter plot showing cilia length in control RPE-1 cells or with CEP55 knockdown (lower, p< 0.0211). **(G)** Representative image of cilia, basal body and Cep55 in Cep55^+/+^ (Wt) MEFs (upper), Cep55^-/-^ (KO) MEFs (middle), Cep55^-/-^ MEFs with ectopic expression of Flag-CEP55 (Rescue) (lower). **(H)** Percentage of ciliated cells in *Cep55*^+/+^, *Cep55*^-/-^ and *Cep55*^-/-^, p< 0.0022; cells reconstituted with a human CEP55 construct (rescue, p< 0.0303). Data represent mean ± SD of 300 cells per genotype (left); scatter plot showing cilia length (μm) in Cep55^+/+^, Cep55^-/-^, p< 0.0001 and CEP55-reconstituted MEFs, p< 0.0101. Data were measured in duplicate across two independent experiments (right). For all calculations, one-way ANOVA (Kruskal–Wallis test) performed, *p < 0.05, **p< 0.01, ***p< 0.001, ****p< 0.0001).

The findings presented above using mouse embryos and human cerebral organoids indicate a role for *Cep55* in neural development. Notably, truncating nonsense mutations in *CEP55* are associated with MKS-like syndrome, a lethal fetal ciliopathy [4,5]. Primary cilia (cilia, hereafter) perform important functions in neurodevelopment, are localized to and extend from RGCs into the lateral ventricle, and are also present in other NPCs and neuron populations [20]. Dysfunction of the ciliary axoneme, basal body, or cilia anchoring structures can all cause defects in cilia organization, leading to ciliopathies [21]. As such, we investigated the involvement of *Cep55* in the regulation of ciliogenesis in the developing neocortex at E14.5 (S3A Fig) and E18.5 (Fig 4A and 4B). We performed immunostaining on embryonic mouse brain sections from *Cep55*^+/+^ and *Cep55*^−/−^ mice using Arl13b (a marker of ciliary membranes), γ-tubulin (basal body), DAPI (DNA marker), and Cep55 to evaluate and compare their expression and localization. We observed fewer cilia in the VZ of *Cep55*^−/−^ brains at E14.5 and E18.5 compared to *Cep55*^+/+^ brain. Our analysis revealed a decrease in both the number and the percentage of ciliated cells throughout the cortical layers at both E14.5 and E18.5, particularly in apical progenitors localized in the ventricle membrane of *Cep55*^−/−^ compared to *Cep55*^+/+^ brains (Figs 4A, 4B and S3A). The decrease in ciliated cells was independent of the reduction in neural progenitor populations (Tbr2+ IPC and Pax6+ RGC) as ciliated cells were expressed as a ratio of cell numbers (S3B Fig). Given the important role of cilia during neurodevelopment, we further examined the potential role of *Cep55* in regulation of ciliogenesis in an *in vitro* model.

### Cep55 is localized to the ciliary basal body and regulates cilia growth

Finding the cilia defect in *Cep55*^*-/-*^ brains, we aimed to determine how Cep55 might regulate ciliogenesis and whether ciliary defects could be recapitulated in an *in vitro* system to facilitate mechanistic studies. We turned our attention to a cell line of neural origin, SH-SY5Y [22], and to the hTERT RPE-1 line, which has been routinely used to study cilia formation [23]. Ciliogenesis typically occurs in G_0_ and G_1_ and serum starvation is widely used to arrest cells in G1 to stimulate cilia formation. Examining the impact of CEP55 knockdown on cilia formation in serum starved SH-SY5Y and RPE-1 cells, we observed significantly decreased numbers of ciliated cells compared to the respective control; the latter also revealed a significant reduction in cilia length after *CEP55* knockdown (Figs 4C, 4D, 4E, 4F, S3C and S3D). Finally, to facilitate rescue studies, we isolated mouse embryonic fibroblasts (MEFs) from *Cep55*^*+/+*^ and *Cep55*^*-/-*^ mice (S3E Fig) to examine possible cilia defects and whether ectopic expression of CEP55 in *Cep55*^*-/-*^ MEFs can rescue the defects. We found that *Cep55*^-/-^ cells had a significantly reduced number of ciliated cells and shorter cilia when compared to *Cep55*^*+/+*^ MEFs (Fig 4G and 4H). In addition, a slight increase was seen in the number of *Cep55*^*-/-*^ MEFs displaying multiple small cilia extending from the basal body (double cilia), alongside a significant proportion of cilia from *Cep55*^*-/-*^ MEFs exhibiting dissociation from the basal body (remnant cilia) (S3F Fig). Notably, most cells with multi-cilia had one nucleus; therefore, multi-cilia are unlikely to be due to failed cytokinesis. Furthermore, we observed that ectopic *CEP55* expression in *Cep55*^-/-^ MEFs (S3G Fig) was able to restore cilia formation and length to levels comparable to *Cep55*^+/+^ MEFs, confirming that defective ciliogenesis was a consequence of Cep55 loss (Fig 4G lower panel and 4H). Given an apparent role for Cep55 in ciliogenesis regulation, we next examined whether Cep55 co-localizes with the cilia axoneme or at the base of cilia by super-resolution microscopy. CEP55 (yellow) was imaged with Arl13b (cilia axoneme marked green) and γ-tubulin (a component of the basal body protein complex marked red) across a population of *CEP55* expressing MEFs. Interestingly, we observed co-localization of CEP55 with gamma-tubulin at the base of cilia (S3H Fig). Together, these findings illustrate that Cep55 localizes at the base of cilia, possibly as a component of the basal body protein complex and is required for normal cilia formation.

### *Cep55*^*-/-*^ MEFs exhibit multinucleation and cell cycle defects

Given that *Cep55*^*-/-*^ embryos are growth restricted *in vivo*, we next sought to recapitulate this phenomenon *in vitro* to determine if Cep55 loss causes proliferation defects in our mouse embryonic fibroblast (MEF) cell lines. We calculated cell doubling time and found significant growth defects in *Cep55*^*-/-*^ lines (S4A Fig) compared to *Cep55*^*+/+*^, which was further revealed to be dose-dependent (S4B Fig), thus indicating *in vivo* growth restriction. Moreover, we were able to rescue this proliferation defect by ectopically expressed *CEP55* in *Cep55*^*-/-*^ MEFs (S4C Fig). Overall, the *in vitro* proliferation deficiency in *Cep55*^*-/-*^ MEFs was consistent with the observed phenotype in neuronal progenitors, where decreased proliferation was detected with Ki67 (E14.5) by immunofluorescence. Furthermore, the multinucleation seen in MEFs (S4D and S4E Fig) was reminiscent of the neuronal phenotype. We next performed cell cycle analysis using propidium iodide (PI)-stained cells sorted by flow cytometry. FACS analysis revealed significant differences in the cell cycle profile of *Cep55*^+/+^ and *Cep55*^-/-^ primary MEFs, where *Cep55*^-/-^ cells showed enrichment of cells in G2 and a reduction in G1 population (S4F and S4G Fig). To further examine cellular division, we performed live-cell imaging of *Cep55*^*+/+*^ and *Cep55*^*-/-*^ cells transduced with mCherry-histone H2B by EVOS-FL time-lapse microscopy (S4H Fig). We found extended cell division (mitotic length) in *Cep55*^*-/-*^ compared to *Cep55*^+/+^ lines (S4I Fig). As expected, the *Cep55*^-/-^ cells showed defective cytokinesis, taking longer to divide effectively with 17% remaining multinucleated (S4J Fig). For further characterization of additional mitotic defects, we performed high-resolution time-lapse microscopy of mCherry-histone H2B cells using confocal microscopy to quantitate mitotic defects including anaphase bridge formation, lagging chromosomes, and mitotic slippage. Although there was a trend towards an increased proportion of anaphase bridges during mitosis in *Cep55*^*-/-*^ MEFs compared to control, this was not statistically significant (S4K Fig). Moreover, we did not observe any changes in lagging chromosomes or slippage (S4K Fig). Together, these results illustrate that Cep55 is important to support normal cell growth and division in particular cytokinesis in MEFs.

### Cep55 regulates Gsk3β, downstream of the Akt pathway

**Fig 5:**
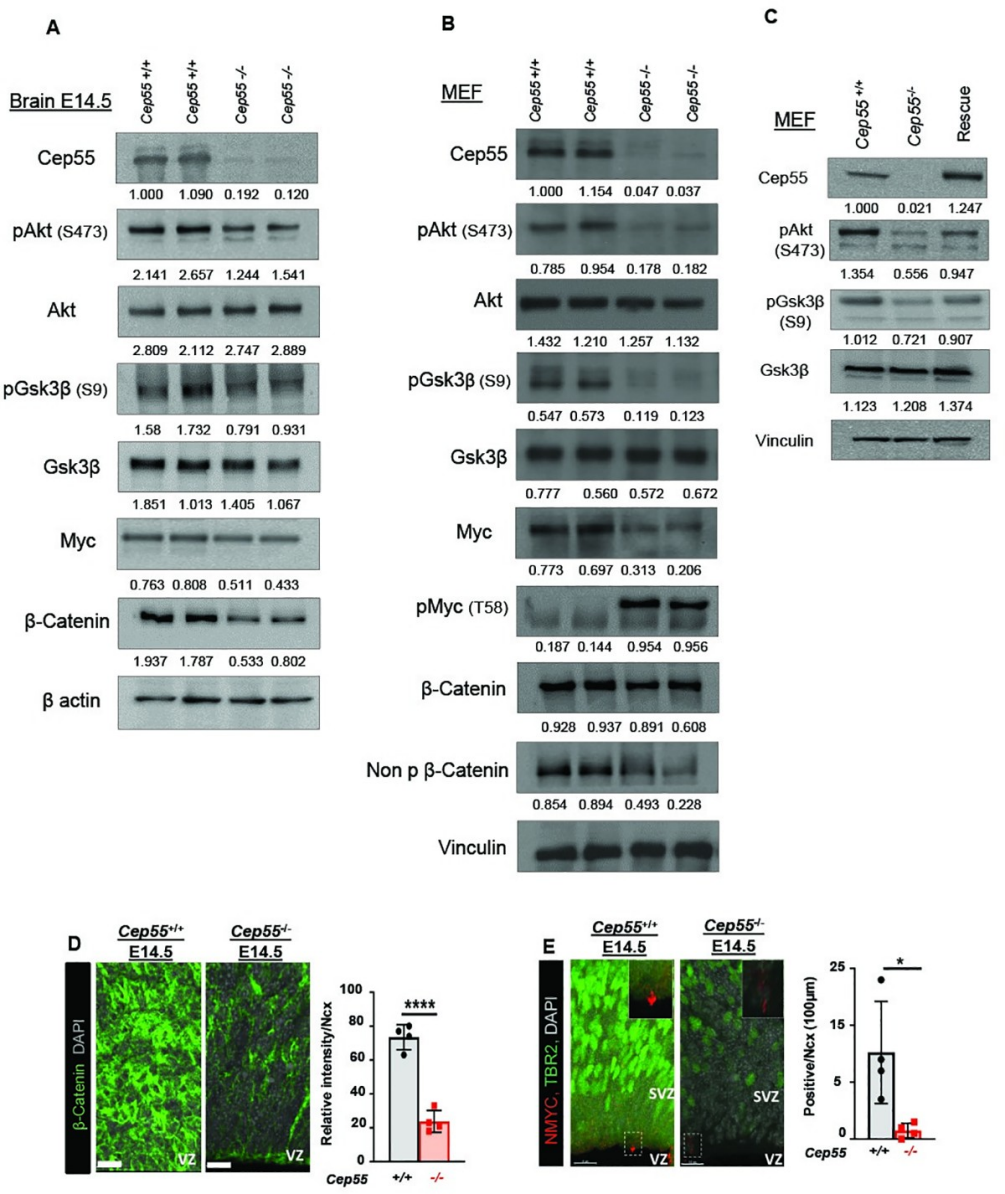
Cep55 regulates GSK3β, β-Catenin and Myc downstream of the Akt pathway. **(A-C)** Representative image of immunoblot (WB) analysis with indicated antibodies. Numbers at the bottom of each WB lane represent the relative quantification of band intensities normalized to the signal of each loading control. β actin or vinculin served as a loading control. Immunoblot performed to compare **(A)** *Cep55*^+/+^ and *Cep55*^-/-^ mouse embryonic brain at E14.5. **(B)** *Cep55*^+/+^ and *Cep55*^-/-^ MEFs. **(C)** *Cep55*^+/+^ (Wt), *Cep55*^-/-^ (KO) and *Cep55*^*-/-*^ MEFs with ectopic expression of CEP55 (Rescue) with indicated antibodies. **(D)** Representative images of *Cep55*+/+ (left) and *Cep55-*/- (right) neocortices stained for β-catenin and nuclei (DAPI) in a 100 μm-width box. Bar chart shows the relative intensity of β-catenin signals for *Cep55*+/+ and *Cep55*-/- neocortices, p< 0.0001; Scale = 50 μm. **(E)** Representative images of *Cep55*+/+ (left) and *Cep55*-/- (right) neocortices stained for N-Myc (red), Tbr2 positive cells and nuclei (DAPI) in a 100 μm-width box. Bar chart shows the percent of N-Myc positive cells, p< 0.0232, scale = 15μm (Mean ± SD of four embryos duplicate technical repeats, Student’s t-test, *p< 0.05, **p< 0.01, ***p< 0.001, ****p< 0.0001).

CEP55 has previously been shown to regulate PI3K/AKT signaling pathway in cancer cells [2,3]. As such, we next performed signaling analysis of Akt and its downstream targets in E14.5 mouse brains as well as in MEFs by immunoblotting. The loss of Cep55 culminated in decreased phosphorylation of Akt (pS473-Akt) in both *Cep55*^-/-^ brain tissue and MEFs (Fig 5A and 5B). Akt controls steady-state levels of Gsk3β through phosphorylation of residue Serine 9 (pS9-Gsk3β). Inactive AKT is known to result in decreased pS9-GSK3β levels, which leads to GSK3β activation with pro-apoptotic functions [24]. In line with this, we observed that Cep55 loss led to the inactivation of Akt (decreased pS473-Akt) and activation of Gsk3β (decreased levels of pS9-Gsk3β) (Fig 5A and 5B). Importantly, we were able to rescue the phosphorylation of Akt and Gsk3β by ectopic expression of *CEP55* in *Cep55*^-/-^ MEFs, confirming the specificity of the observed signal transduction effects (Fig 5C). Activated Gsk3β has been shown to inhibit downstream targets involved in proliferation such as β-catenin and Myc. We observed a decrease in β-catenin levels in E14.5 *Cep55*^*-/-*^ brains (Fig 5A). Similarly, we observed reduced β-catenin and non-phospho β-catenin levels in *Cep55*^*-/-*^ MEFs (Fig 5B). GSK3β can also destabilize MYC by phosphorylation on Threonine 58 [25]. Accordingly, we observed an increase in pT58-Myc levels and a concomitant decrease in total Myc levels in *Cep55*^*-/-*^ MEFs as well as a trend towards decreased total Myc levels in E14.5 brains when compared to *Cep55*^*+/+*^ controls (Fig 5A and 5B). Additionally, we performed IHC staining of total β-catenin and N-Myc on E14.5 brain sections from *Cep55*^*-/-*^ and *Cep55*^*+/+*^ embryos. These results revealed a significant decline of membranous and cytoplasmic β-catenin in *Cep55*^-/-^ in the VZ compared to *Cep55*^+/+^ in E14.5 brain, consistent with immunoblot analysis in the embryonic brain at this time-point (Fig 5D). Similarly, IHC on E14.5 brain sections revealed a significant reduction in N-Myc expression in *Cep55*^*-/-*^ NPCs compared to *Cep55*^+/+^ controls (Fig 5E). Notably, *Cep55* loss resulted in a reduction in transcript levels of Myc in both brain samples and MEFs, consistent with reported transcriptional regulation of Myc by the Wnt/β-catenin pathway (S5A and S5B Fig). Additionally, N-Myc (a member of the Myc family regulating neural cells) was shown to be reduced in E14.5 *Cep55*^*-/-*^ brain tissue (S5A Fig). Together, we conclude that Cep55 loss potentially inhibits proliferation and survival in an Akt-dependent manner.

### Activation of PI3K/AKT signaling pathway rescues the proliferation and cilia defects induced by *Cep55*-loss

**Fig 6:**
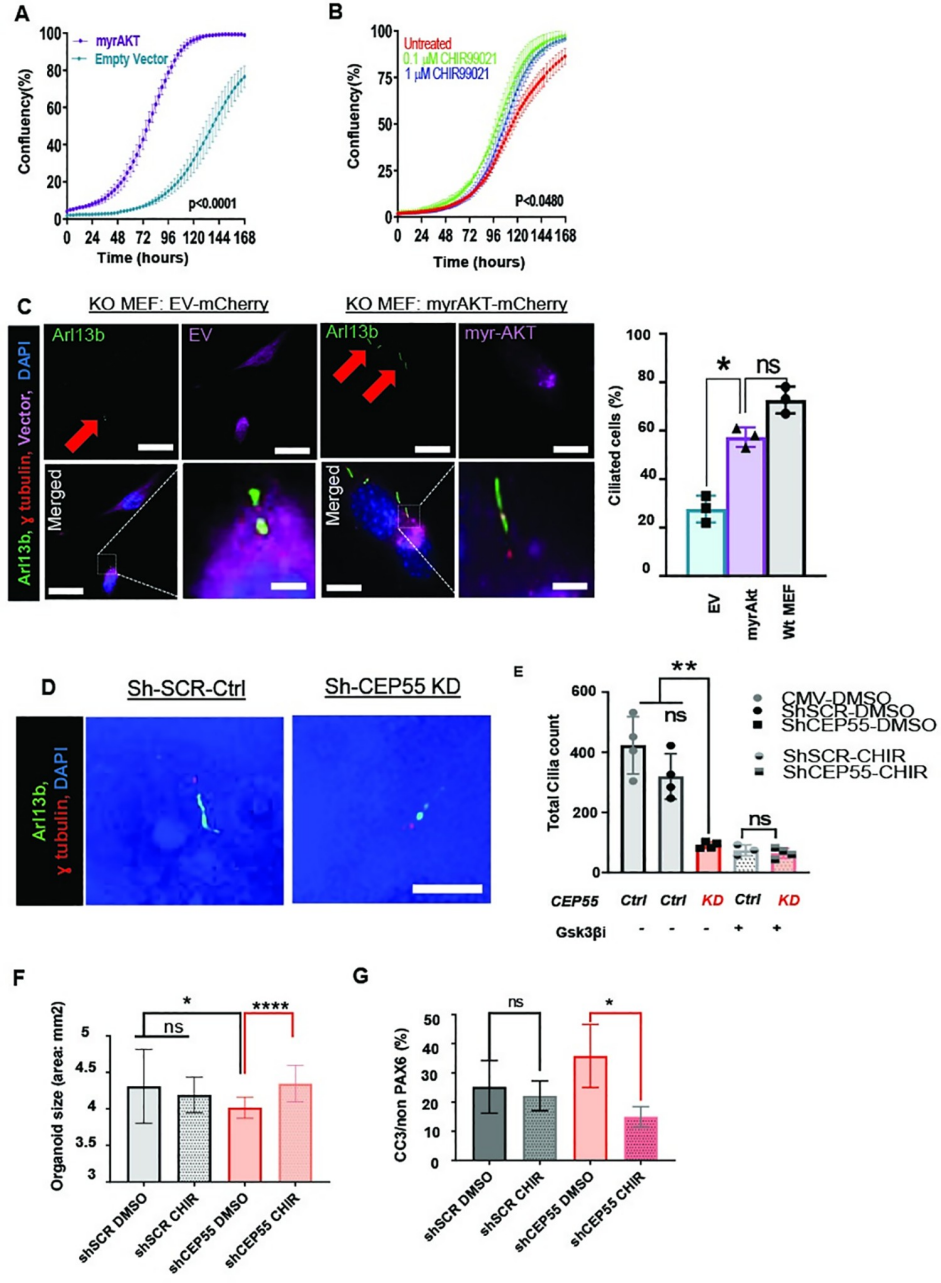
Myr-AKT and downstream effectors can rescue *Cep55* loss. Proliferation assay showing the growth of *Cep55*^-/-^ (KO) MEF **(A)** Transiently transfected with EV (turquoise blue) or myr-AKT (purple) and **(B)** treated with indicated doses of GSK3β inhibitor, CHIR99021 (untreated: red, 0.1 μM inhibitor: green, 1 μM inhibitor: blue). Mean ± SD, the average of 2 biological repeats and 3 independent experiments Student’s t-test, ****p< 0.0001. **(C)** Representative images of *Cep55*^-/-^ (KO) MEFs reconstituted with EV-mCherry (left) or myrAKT-mCherry (right) immunostained for cilia (Arl13b), basal body (Ɣ-tubulin) and nuclei (DAPI). The lower panel shows merged images with magnification of the boxed area. Scale = 12 μm. Left: Percentage of ciliated cells in *Cep55*^+/+^ (Wt) and *Cep55*^-/-^ (KO) MEFs transfected with EV or myr-Akt. Mean ± SD, n = 100 cells from 3 independent experiments Student’s t-test, *p< 0.05). **(D)** Representative image U6 (shSCR Ctrl) and CEP55 KD human brain organoids immunostained for cilia (ARL13b), basal body (Ɣ-tubulin) and nuclei (DAPI), Scale = 10 μm. **(E-G)** Comparison of U6 (shSCR Ctrl) and CEP55 KD untreated (DMSO) and treated with 3 μM GSK3β inhibitor, CHIR99021 for **(E)** Ciliated cell counts, p< 0.0010 **(F)** The size of organoids (area) p< 0.0380 for control, p< 0.0001 for shCEP55 **(G)** The percent of cleaved Caspase 3 in PAX6 negative cells, p< 0.5767 for control, p< 0.0104 for shCEP55. Data were measured across two independent experiments. n = 6 organoids. Mean ± SD, one-way ANOVA (Kruskal–Wallis test) performed, **p< 0.01).

Next, we sought to evaluate whether the reconstitution of Akt signaling or its downstream regulators would be sufficient to rescue the proliferation and ciliogenesis defects in *Cep55*^*-/-*^ MEFs. To investigate this, we first, utilized a myristoylated form of AKT1 (myrAKT) previously described to be constitutively-active [26]. *Cep55*^-/-^ MEFs were transduced with retrovirus to express myr-AKT or an empty vector (EV) control (S5C Fig) and assessed for proliferation. Incucyte analysis revealed that myr-Akt was able to markedly increase the proliferative rate of *Cep55*^*-/-*^ MEFs when compared to EV-transduced cells (Fig 6A). We also sought to determine if we could rescue the proliferation and ciliogenesis defects using an inhibitor of activated GSK3β. The universal GSK3β inhibitor, CHIR99021, at low dosages (0.1 μM and 1 μM) was able to partially increase proliferation in *Cep55*^-/-^ MEFs (Fig 6B), possibly through the inhibition of active GSK3β as per previous reports [27]. In contrast, in *Cep55*^+/+^ lines (similar to *CEP55* rescued lines where Gsk3β is inactivated by Akt activity); Gsk3β inhibition can hinder proliferation in a dose-dependent manner (S5D Fig). Our findings demonstrate that Cep55, through activation of Akt and inhibition of Gsk3β, can regulate proliferation.

**Fig 7:**
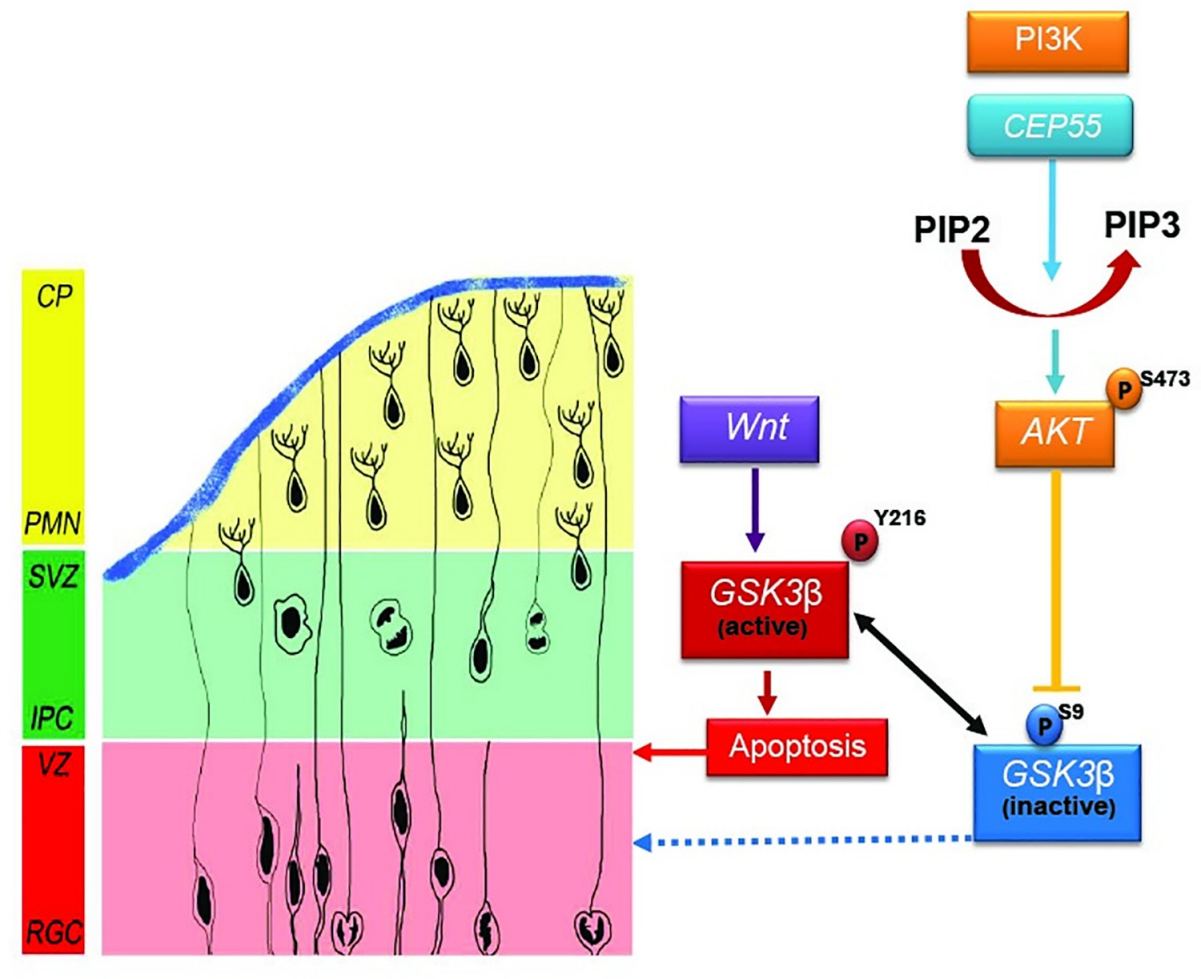
Graphical abstract. Proposed model of Cep55-dependent regulation of RGC proliferation or apoptosis through PI3K/Akt and the downstream targets Gsk3β, β-Catenin and Myc. CEP55 is known to bind the catalytic subunit of PI3K (p110) and promotes downstream phosphorylation of AKT (S473). The active AKT inactivates GSK3β by phosphorylating it on S9. However, in Cep55 KO cells, downregulation of Akt phosphorylation leads to upregulation active Gsk3β (Y216) and consequent cell death which can be rescued by Gsk3β inhibition. Ciliogenesis is regulated in an Akt-dependent manner and independent of Gsk3β.

Next, we examined whether myrAKT expression was sufficient to rescue the defects in cilia formation. Strikingly, we observed that myrAKT expression in *Cep55*^*-/-*^ MEFs, but not EV expression, restored the percentage of ciliated cells to levels more comparable to *Cep55*^+/+^ MEFs (Fig 6C). Regarding ciliogenesis, inhibition of Gsk3β in *Cep55*^*-/-*^ MEFs did not affect cilia formation significantly (S5E Fig). Our analysis of cilia in *CEP55* KD organoids revealed that CEP55 loss perturbed ciliogenesis (Fig 6D). However, consistent with our MEF data, we were unable to rescue this phenotype with GSK3β inhibitor (Fig 6D and 6E). This is consistent with a previous study that showed that GSK3β inhibition alone does not modulate ciliogenesis but combined inactivation of Von Hippel-Lindau (VHL) and GSK3β leads to loss of cilia formation and maintenance [28] suggesting that GSK3β acts redundantly with VHL to regulate ciliogenesis. Strikingly, GSK3β inhibition can rescue the size of human organoids. CEP55 loss in organoids led to size (area) decrease compared to control, consistent with microcephaly seen in CEP55-null human patients and mouse model (Fig 6F). While the CHIR99021 (GSK3β inhibitor) treatment had no significant effect on the size of control organoids, it led to a significant increase in the size of *CEP55* KD organoids (Fig 6F). The mechanism of this rescue is through the reduction of apoptotic cells as marked by cleaved caspase 3 (Fig 6G). Taken together, our findings demonstrate that CEP55 regulates cell survival in an AKT-dependent manner (Fig 7). Nevertheless, CEP55-dependent regulation of ciliogenesis might occur through an AKT downstream effector(s) independent from GSK3β. Overall, the phenotype in brain organoids and related apoptosis can be rescued through GSK3β inhibition.

## Discussion

CEP55 was initially described as an abscission component serving to regulate cellular segregation during cytokinesis [1]. Later, the finding of CEP55 regulatory roles in PI3K/AKT survival signaling illustrated the importance of this protein, especially in cancer where transcriptional upregulation of *CEP55* widely contributes to cancer progression [2,29]. Interestingly, activating mutations in genes of the PI3K pathway has been shown to cause a wide range of brain and body overgrowth disorders [30] with phenotypic severity highly dependent on the extent of activation of the pathway [31]. In contrast, the reduction in the activity of the PI3K pathway in specific organs can lead to decreased organ size [32]. Recently, homozygous nonsense *CEP55* mutations that cause truncated protein products have been linked to lethal fetal syndromes, demonstrating the importance of *CEP55* in embryogenesis and especially in neuronal development [4–6]. Surprisingly, patients that are compound heterozygotes for nonsense and missense, or splicing mutations, in CEP55 survive [33]. However, to date, the exact molecular mechanism underlying these disorders has remained elusive. By simultaneously studying *Cep55*^-/-^ mouse model and human cerebral organoids, our studies provide novel insights into the pathophysiological role of *Cep55* to understand disease linked to dysregulation of this gene. The neural phenotypes in this model, including severe microcephaly with diminished cortical cellularity overlap with the human *Cep55-associated* disorders. Notably, we also observed a higher proportion of multinucleated neurons in E14.5 *Cep55*^*-/-*^ brains when compared to controls, reminiscent of that observed in infants affected by MARCH syndrome [6]. Consistently, a significant proportion of immortalized MEFs also exhibited multinucleation upon loss of Cep55.

The brain size of *Cep55-*deficient embryos is significantly reduced compared to controls due to hypocellularity. It is conceivable that the apoptosis seen in brain sections could progress to a major loss of cerebral hemisphere parenchyma, resulting in marked cavitation, leaving only a small amount of residual cortical tissue, and compensatory expansion of the lateral ventricles (termed hydranencephaly: seen in human with CEP55 mutation), or porencephaly, if the cystic change and parenchymal loss was less severe. For the detailed characterization of neurodevelopmental defects upon loss of *Cep55*, in this study, we have concentrated on the development of the neocortex. Analysis of the overall distribution of neurons across the neocortex revealed a decreased population of all NPCs and neurons in *Cep55*^*-/-*^ brains including RGCs, IPCs, and PMNs. A deficiency in cell proliferation in *Cep55*^-/-^ brains at early neurodevelopment stages, as well as the significant increase of apoptosis in both NPCs and PMNs, reinforce a pro-survival role of Akt activation, which is significantly compromised in *Cep55*^-/-^ brains. This finding is in line with the time-lapse data showing that cell death is not due to the mitotic catastrophe caused by aberrant cytokinesis but rather cells are mainly dying during interphase. Our data indicate the presence of proliferation defects associated with loss of Cep55 in E14.5 embryo brains, as well as *in vitro* in MEF models with both constitutive and conditional Cep55 loss. This proliferation defect could be rescued in *Cep55*^-/-^ MEFs by ectopic expression of *CEP55*. Overall, the *in vitro* proliferation deficiency in *Cep55*^-/-^ MEFs was consistent with the observed phenotype in neuronal progenitors, with decreased proliferation observed by Ki67 immunohistochemistry at E14.5. We also observed increased levels of cleaved caspase-3, a marker of apoptotic cell death by immunoblots in *Cep55*^-/-^ embryonic brain tissue consistent with increased apoptosis as assessed by TUNEL staining of brain sections by IHC.

During the preparation of this manuscript, three reports [8–10] demonstrated generation of viable homozygous *Cep55* KO pups at P0 whereas in our study *Cep55* KO were very rarely born (1/29 at P0). Tedeschi et al. [8] and our *Cep55* knockout mice were generated using ES clones from the European mutant mice consortium, albeit using different clones (HEPD0726_6_A04; HEPD0726_6_B01). Both studies generated a *tm1a* allele, in C57BL/6 genetic background, but there could be substrain differences, whereas the other two studies generated mice using CRISPR-Cas9 in either a mixed C57BL/6 and FBV/N or pure C57BL/6 background [9,10]. These prior studies, also reported slight differences in postnatal lethality ranging from preweaning lethality with incomplete penetrance, ~7% homozygous mice viable between (P1- P14) to 100% mortality for KO pups at P2 [8,9]. We observed a significant reduction in the numbers of KO pups obtained at P0 compared to the expected number based on Mendelian ratio. This difference in P0 survival may be explained by genetic difference in substrain and different *Cep55* ES *tm1a* clone used by us and Tedeschi et al. [8]. Differences in the approach used to target *Cep55* in the other two studies may also contribute to this discrepancy [9,10]. The different genetic backgrounds are known to have major effects on the penetrance and severity of phenotypes. As an example, in *Tcof1* mouse model the offspring exhibit markedly variable strain-dependent phenotypes that widely range from extremely severe and lethal to apparently normal and viable mice [34]. The environmental differences between facilities (diet, bedding, light, noise and disruption etc) may also affect perinatal viability either directly on health of the pup or indirectly through impact on maternal care.

Importantly, however, there are similarities in our report and previous studies, where Cep55^-/-^ animals fail to thrive and consistently perish around the time of birth or soon after. All studies report malformation of brain, microcephaly, decrease in length, thickness, and cellularity of cortex. We and others also report reduction in neuronal stem and progenitor cells and accumulation of binucleated neurons and increased apoptosis in brain. Tedeschi et al. [8] suggested cell type specific requirement of Cep55 and endosomal sorting complexes required for transport (ESCRT) in control of cell abscission, which is a known function of Cep55 [35]. This was observed in NPCs but not in primary fibroblasts, suggesting a CEP55-ESCRTIII-independent mechanism of abscission control in non-neural cells. Notably, primary fibroblasts were shown to undergo cytokinetic abscission in the absence of Cep55 or after depletion of ESCRTIII. In contrast, Little et al. [10] reported alternative findings demonstrating that ESCRTs recruitment is not absent but rather reduced in Cep55-null cells, suggesting that there is not an absolute requirement of Cep55 for recruitment of ESCRTs. A role for Cep55-ESCRT in regulation of non-neural cell division has also been reported [10], refuting the claim of the aforementioned study [8] that Cep55 is dispensable for non-neural cell division. Notably, the authors observed contrasting p53 apoptotic responses to cytokinetic defects in different cell types and suggested it as a potential mechanism to explain disparate tissue phenotypes[10]. P53 accumulated in *Cep55*^*-/-*^ cortical cells and p53 KO partially rescued the microcephaly phenotype in *Cep55*^*-/-*^ mice, but cortical disorganization still occurred.

The present study provides evidence that cytokinetic and abscission defects due to loss of Cep55 do not explain increased apoptosis of NPCs, as in *Cep55*^*-/-*^ neocortices and in knockdown human brain organoids, the apoptotic NPCs were frequently mononucleated, suggesting that loss of prosurvival role of Cep55, possibly due to reduced PI3K/Akt pathway activation in absence of Cep55, might result in cell death. This is consistent with our prior study in cep55 mutant Zebrafish [36]. Notably, the cytokinetic defect in *Cep55* mutant Zebrafish was very mild and the microcephalic phenotype was attributed to increased apoptosis. Previous studies on brain organoids generated from pluripotent stem cell lines with defects in primary microcephaly specific genes including *ASPM* [37], *CPAP* [38], *WDR62* [39] and *CDK5RAP2* [40] suggested that a loss in NPCs was caused by their premature differentiation, not by apoptosis, that lead to depletion of the progenitor population [41–43]. Our study shows that reduction in NPCs in CEP55-depleted human organoids was caused by increased cell death, which holds true for our *Cep55* KO mouse model and those reported by others [8–10].

Additionally, our study documents a novel contribution of hyperactive Gsk3β to the observed neural phenotype. The dysregulation of Akt [44] as well as its downstream effectors such as GSK3β [45], Myc, and β-Catenin [46] have been reported to have adverse effects on neurodevelopment. GSK3β, implicated as a master regulator of NPCs, is a central mediator of a wide range of processes in neurodevelopment [47]. Our data showed reduced Akt phosphorylation and a consequent reduction in inhibitory phosphorylation on Gsk3β in the absence of Cep55 leads to Gsk3β activation and consequent proteasomal mediated degradation of its substrate, β-catenin. In the *Cep55* ^-/-^ brain and MEFs we observed reduced expression of β-catenin by immunoblot and immunohistochemistry in the absence of any changes at the level of transcription. We identified Myc destabilization in MEFs and embryonic brain in protein level by immunoblot and decrease in Myc and N-Myc transcript levels by RT-qPCR. We also validated these results by IHC analysis which revealed a decline in N-Myc protein expressed mostly in the VZ of mouse brains. Overall, reduced proliferation and increased apoptosis in NPCs upon *Cep55* deletion could explain smaller brain size.

Primary microcephaly is caused by autosomal recessive mutations in genes that control the assembly of centrosomes and cilium, and as such, cell lines generated containing mutations in these genes display defects in cell cycle progression, primary cilium formation and viability issues [48]. Multiple pieces of evidence obtained in this study support Cep55-dependent regulation of ciliogenesis. First, ciliogenesis was consistently defective in NPCs from *Cep55*^-/-^ embryonic brain sections (E14.5) and CEP55-depleted human cerebral organoids. Moreover, several other cellular models used in the study including *Cep55*^-/-^ MEFs and *CEP55*-depleted RPE-1 and SH-SY5Y cells also exhibited a primary cilium defect. This indicates that Cep55 is broadly required for cilia formation in multiple mouse and human cell types. This is consistent with the described association of *CEP55* with human MKS like ciliopathy syndrome [4,5]. Second, we found that *Cep55* is predominantly localized at the base of the primary cilium consistent with a function in the assembly or anchoring of cilia axoneme. Third, myr-AKT overexpression is sufficient to restore the deficit in cilium length and proliferation defect in Cep55-deleted MEFs. There is emerging evidence that the cilia dysfunctions contribute to many neurogenetic disorders such as Meckel-Gruber syndrome [49]. There is also a known relationship between AKT pathway activation and primary cilia wherein pAKT has been shown to localize to the basal body. Consequently, Akt knockdown can suppress cilia formation [50]. We propose that defective activation of the evolutionary conserved PI3K/Akt pathway in absence of Cep55 leads to defective survival of neurons as well as defective cilia formation. However, inhibition of activated Gsk3β, observed in *Cep55* ^-/-^ MEFs and human organoids as a consequence of reduced Akt activation, could only rescue the phenotype through decreasing apoptosis without any impact on ciliogenesis, suggesting that other downstream effectors of Akt are involved in the regulation of ciliogenesis. Recently it was reported that Cep55 was required for cilia resorption [9]. The absence of cilia formation defect in this study may be a consequence of severity of Cep55 loss between mouse models. Similarly, we have reported substantially more severe post-natal viability of Cep55-/- offspring in our mouse model. Importantly, our current finding that Cep55 is required for cilia formation does not rule out an involvement in cilia resorption. Indeed, several centrosomal proteins such as PLK1 [51], CEP170 [52] and WDR62 [48] have been shown to be involved in both cilia assembly and disassembly at different stages of the cell cycle. However, the severity of ciliogenesis defect in our animal and cell models does preclude us from examining a cilia resorption defect.

In summary, our study employed a mouse model and human brain organoids to identify the critical role of *Cep55* during brain development. We suggest that defective PI3K/Akt pathway activation and consequently, increased apoptosis during embryogenesis could be the predominant cause of microcephaly seen in *CEP55* loss-associated genetic syndromes. In addition, we revealed an important role of Cep55 in regulating ciliogenesis in an Akt dependent manner. Further studies where we can generate inducible CEP55 KO human pluripotent stem cell lines should allow more in-depth evaluation of CEP55 biology in particular to what extent disruption of ciliogenesis contributes to complex CEP55-associated clinical phenotypes [4–6].

## Materials and methods

### Ethics statement

This research was carried out in strict accordance with the Australian Code for the care and use of animals for scientific purposes. All protocols were approved by the QIMR Berghofer Medical Research Institute Animal Ethics Committee (ethics number A0707-606M).

### Generation of constitutive *Cep55* knockout mice

*Cep55* floxed ES cells were purchased from the International Knockout Mouse Consortium (Exon 6 of *Cep55* was trapped, IKMC Project ID:93490) and heterozygous *Cep55* targeted mice were generated by the Australian Phenomics Network (APN) facility, where the targeted allele acts as a gene-trap to form a non-functional (KO) allele. The knockout-first allele used in the targeting strategy is amenable to the generation of a floxed allele via FLP recombinase breeding, allowing the generation of conditional knockout mice. Mice were housed at the QIMR Berghofer Medical Research Animal Facility in OptiMICE caging (Centennial, Colorado, USA) at 25°C with a 12-hour light-dark cycle. All experimental animals were maintained on a C57BL/6J strain.

### Genotype analysis

Genotyping was performed using genomic DNA extracted from mouse ear using the QuickExtract DNA Extraction Solution (Lucigen, USA) according to the manufacturer’s protocol. Wild-type and *Cep55* transgenic alleles were genotyped using a 3-primer PCR with a common forward primer (P1) and two different reverse primers (P2 and P3) to differentiate between different allele forms. Primer sequences were as follows: *Cep55* P1(TGGGTCTTTAACTCATGGTC), *Cep55* P2 (AGGAGTGAAAAGTCCTCACA), *Cep55* P3(GTACCGCGTCGAGAAGTT).

### Quantitative reverse transcription PCR

Quantitative reverse transcription PCR (RT-qPCR) was performed using cDNA as the template with gene-specific primers as outlined below. The reverse Transcription obtained using the SuperScript First-Strand Synthesis System. A control reaction was performed without reverse transcriptase to ensure no genomic DNA had contaminated the samples, as well as a no-template DNA control. The RT-qPCR was performed in 96-well plate format using a SYBR Green master-mix (Roche Applied Science, Basel, Switzerland) with a CFX96 Touch Real-Time PCR Detection System (Bio-Rad Laboratories, US). The total volume of each reaction was 8 μL including 4 μL of Sybr green, 1 μL of each primer (1 picomole of each), 1 μL of cDNA (10ng) and 2 μL of sterile water. The specificity of RT qPCR amplification was examined by checking the melting curves and running each sample on a 2% agarose gel. The results were analyzed by the ΔΔCt method. Actin was used as a housekeeping gene.

### RT qPCR primers

*Myc* (CGGACACACAACGTCTTGGAA / AGGATGTAGGCGGTGGCTTTT),

*Mycn* (CCTCCGGAGAGGATACCTTG / TCTCTACGGTGACCACATCG),

*Cep55* (AAGGCAGAAGCAGACTCTTGGAGA / GTGGCGGACAGCTGGTTTTTCA and

TCGAGCTGGAAAAGAGAACAG / TGCTTCTCCACTTGAAGATAACC)

### Organ/embryo isolation

Mouse organs were isolated using the Nikon SMZ45 stereo dissecting microscope (Nikon Inc, Tokyo, Japan). The isolated organs were washed in ice-cold Phosphate Buffered Saline (PBS). For protein or mRNA extraction, the samples were snap-frozen on dry ice. For histology staining, tissues were fixed in either the Bouin solution (pathology investigation) or 4% PFA (immunohistochemical staining) for 24–48 hours.

### MEF establishment

MEFs were isolated from E13.5 embryos from *Cep55*^+/−^ inter-crosses for the constitutive MEF. Embryos were dissected into ice-cold sterile PBS, followed by removal of the internal viscera and head for genotyping. The remaining tissue was incubated in trypsin-EDTA (Sigma Aldrich, St Louis, USA) and disaggregated by mechanical shearing using a sterile scalpel blade. The dispersed tissues were further homogenized by trituration and transferred into 25cm^2^ flasks (Corning) and allowed to adhere overnight. Primary MEFs were maintained in Dulbecco’s Modified Eagle’s Medium (DMEM) (Life Technologies, Carlsbad, CA, USA) containing 20% Fetal Bovine Serum (SAFC Biosciences, Lenexa, USA) 1% penicillin-streptomycin (Life Technologies) and 1% Amphotericin B. Primary MEFs prior to passage 5 were used for experiments as indicated. Retroviral SV40 transfection was used for the immortalization of MEFs.

### Cell culture

Mouse embryonic fibroblasts (MEFs) were generated as per extended methods. Retinal Pigment Epithelium (RPE-1) cells were obtained from the Diamantina Institute, UQ. Human neuroblastoma (SH-SY5Y) cell line was cultured in a 1:1 mix of DMEM and F-12 supplemented with NEAA (1%, non-essential amino acids), FCS (10%) and pen/strep (100 U/ml). Pluripotent stem cell line HES3 (WiCell) was maintained in mTeSR1 media (StemCell Technologies) and passaged every 4 days using ReLeSRTM as per manufacturer’s instructions (StemCell Technologies) and reseeded at 12,000 cells per cm^2^ onto T25 cell culture flasks coated with Matrigel (Corning). All the cell lines were routinely tested for Mycoplasma infection by Scientific Services at QIMR Berghofer Medical Research Institute.

### Cerebral brain organoid differentiation

Cerebral brain organoids were generated from single-cell dissociated HES3 pluripotent cultures according to previously described methods [53–55]. Briefly, single-cell suspensions were counted and seeded at 10^4^ cells per well of U bottom 96 well ultra-low attachment plates (Corning) in mTeSR1 media supplemented with 10 μM ROCK inhibitor and centrifuged at 300g for 3 minutes to allow for initial aggregation. The following day, media in each well was replaced with knockout serum replacement (KSR) media, consisting of DMEM/F12, 20% KSR, 1x Penicillin/Streptomycin, 1x Glutamax, 1x Non-essential amino acids and 0.1mM β-mercaptoethanol (Life Technologies). KSR media was supplemented with 2 μM Dorsomorphin and A83-01 (Sigma-Aldrich) and changed daily for the first 5 days of induction. Between days 5 and 6 of induction, media was changed at a 1:1 ratio with neural induction media consisting of DMEM/F12, 1x N2, 1x Glutamax, 1x Non-essential amino acids, 1x Penicillin/Streptomycin and 10μg/mL Heparin (Stem Cell Technologies) supplemented with 1 μM CHIR99021 and SB-431542 (Sigma-Aldrich). At day 7, media changes consisted only of neural induction media supplemented with 1 μM CHIR99021 and SB-431542 until day 14, at which point cultures were changed to neural differentiation media consisting of 1:1 base media of DMEM/F12 and Neurobasal, 1x Glutamax, 1x Non-essential amino acids, 1x N2 and B27 (with vitamin A) supplements, 1x Penicillin/Streptomycin, 0.05mM β-mercaptoethanol and 2.5μg/ml insulin (Life Technologies).

### Doubling time assay

MEFs were plated in a 10 cm petri dish, at a density of 10^5^ cells per well, in triplicate for each genotype. Every second day, cells were collected, and the overall cell number assessed using a Countess automated cell counter (Life Technologies) for a total of 6 days.

### Cell proliferation assay

Cells were seeded at a density of 5×10^3^ or 10^4^ cells per well in duplicate, and growth assessed using an IncuCyte S3 Live-Cell Analysis system (Essen BioSciences Inc, USA) Where treatments were performed, drugs were added the day following cell seeding.

### Cell cycle analysis

Cells were plated in a 6-well plate in duplicate at a density of 10^5^ cells per well and harvested in trypsin-EDTA (Sigma Aldrich, St Louis, USA) at indicated time points and fixed in ice-cold Ethanol for 24 hours. Cells were stained in 1mg/mL of propidium iodide (Sigma Aldrich) and 15mg/mL RNAse A) at 37°C in the dark. DNA content was assessed using a FACScanto II flow cytometry (BD Biosciences, Mountain View, CA). The proportion of cells in G0/G1, S phase and G2/M were quantified using ModFit LT 4.0 software (Verity Software House, Topsham, ME, USA).

### Live-cell imaging and microscopy

Live-cell imaging was performed on an EVOS Fl Auto (ThermoScientific) or Spinning disk confocal (Andor) microscope using MetaMorph Microscopy automation and image analysis software. Images were analyzed using analySIS LS Research, version 2.2 (Applied Precision).

### Histopathological analysis and immunohistochemistry staining

For histopathologic investigation with hematoxylin and eosin (H&E), tissues were collected and fixed Bouin’s solution (Sigma-Aldrich, USA) for 48 hours and embedded in paraffin blocks. 5 μm–thick sections were prepared for H&E staining with a Leica Autostainer XL. For periodic acid–Schiff (PAS) staining, tissues were removed from mice and fixed in 4% PFA for 24–48 hours. Tissues were embedded in paraffin and wax-embedded tissues were sectioned at 5–10 μm and mounted on Superfrost Plus slides (Thermo Fisher Scientific) using the Sakura Tissue-Tek TEC (Sakura Finetek, Tokyo, Japan). The slides were then dewaxed and rehydrated by standard protocols.

Antigen retrieval protocol which was performed with 10 mM Sodium citrate buffer pH = 6.0 using a Decloaking Chamber NxGen (Biocare Medical, USA) for 15 minutes at 95°C. Sections were permeabilized and blocked in blocking buffer at RT for at least 1 hour (20% FBS / 2% BSA / 0.2% Triton X-100 in PBS. Primary antibodies were diluted in blocking buffer and incubated at 4°C overnight in a humidified chamber. Alexa-fluor-conjugated (Life Technologies) secondary antibodies were incubated at RT for 3 hours in a humidified chamber. Slides were mounted with Vectashield (Vector Laboratories, Burlingame, CA, USA) followed by cover-slipping using a Leica CV5030 (Leica Biosystems, Wetzlar, Germany) glass coverslipper and Shandon Consul-Mount mounting media (Life Technologies). Slides were scanned with Aperio Scanscope FL/XT (Aperio, Vista, USA) using 20X or 40X magnification and imaged with an LSM780 confocal microscope (Zeiss, Jena Germany) before analyzing with Image Scope software (Leica Biosystems, Buffalo Grove, IL, USA). Nuclei count v9 algorithm or Imaris (Bitplane Scientific Software, Belfast, United Kingdom) was used to score immuno-positive cells.

### Immunofluorescence (IF)

For immunofluorescence (IF) assays, cells were counted and seeded at 5×10^4^ cells on sterile glass coverslips. For assessing cilia, MEFs were serum-starved for 48 hours prior to analysis. Where indicated, drugs were added 24 hours prior to fixation. Cells were fixed in 4% PFA (Sigma Aldrich, St Louis, USA) or ice-cold Methanol (100%) for the centrosomal protein in PBS for 20 minutes at RT and permeabilized in 0.1% TritonX-100 (Sigma Aldrich) for 10 minutes or 90 seconds (cilia experiments) and blocked in 3% or 1% (cilia experiments) bovine serum albumin (BSA; Sigma Aldrich) in PBS for 1 hour in a humidified chamber at RT. Coverslips were washed and incubated with Alexa-fluor-conjugated secondary antibodies (Sigma Aldrich) diluted in 3 or 1% BSA (1:1000) for 30 minutes at 37°C in a humidified chamber in the dark. Coverslips were mounted using Prolong gold anti-fade mounting medium (Life Technologies). Imaging was performed on a DeltaVision personal DV deconvolution microscope and DeltaVision Ultra (super-resolution) (Applied Precision, GE Healthcare, Issaquah, WA) and analyzed using the GE DeltaVision software package. Automated counting was performed using script modules of Fiji-ImageJ software (Java3D, Minnesota, USA).

### Whole-mount immunostaining

Brain organoids were stained following a previously published protocol [56]. Briefly, organoids were fixed in 1% paraformaldehyde solution overnight at 4°C. After washing, organoids were incubated for 4 hours at room temperature in a blocking buffer consisting of 5% FBS and 0.2% Triton X in PBS. Organoids were incubated with primary antibodies (extended methods) in blocking buffer overnight at 4°C, followed by washing in blocking buffer and subsequently incubated with secondary antibodies and Hoechst (1:1000) overnight at 4°C. Organoids were again washed twice in blocking buffer at 4°C and subsequently mounted onto microscope slides using Prolong glass antifade mountant (Life technologies). Live imaging was carried out using an Andor WD Revolution spinning disk microscope to assess an increase in integrated viral GFP. Immunostained samples were imaged using a Zeiss 780-NLO confocal microscope. Four random fields of view were imaged per organoid and manually quantified using Fiji software.

### Antibodies for immunostaining

Immunostaining was performed with the following primary antibodies: Ki67 1:500 (rabbit, NCL-ki67p; Novacastra, Wetzlar, Germany); mouse anti γ-tubulin (1:400, T5326 Sigma), rabbit anti γ-tubulin (1:400, T5192 Sigma), rabbit anti Arl13b (1:300, 17711-1-AP Proteintech), mouse anti α-tubulin (1:300, T5168 Sigma), mouse anti Arl13b (1:300, 75287, Antibodies Inc.), rabbit anti TBR1 (1:200, ab31940 Abcam), Monoclonal Anti-Acetylated Tubulin antibody produced in mouse clone 6-11B-1(Sigma Aldrich), rat anti TBR2 488 (1:200, 53-4875-80 eBioscience), rabbit anti phospho-histone3 (1:300, ab47297 Abcam), mouse anti PAX6 (1:200, DSBH), Cep55 (1:500; sc-374051Santa Cruz biotechnology), Pericentrin (1:1000; Covance, PRB-432C), β-Catenin (1:1000; Cell Signaling Technology, 9582), Cleaved Caspase-3 (1:500; 9664 Cell Signaling Technology), Tuj1 (TU20) (1:200, 4466s Cell Signaling Technology), GFP (1:500, AB290 Abcam), DAPI was used for the nuclear staining (D9564; MilliporeSigma). ApopTag staining was performed with an ApopTag peroxidase in situ apoptosis detection kit (S7100; MilliporeSigma, Billerica, MA, USA).

### β-Galactosidase staining

Detection of β-Galactosidase Activity using LacZ reporter and X-gal Staining was performed as described by (Burn, 2012). X-gal (5-Bromo-4-chloro-3-indoxyl-beta-D-galactopyranoside, GoldBio) was used to detect reporter gene expression marked by a dark blue stain. Briefly, whole embryos/organs were dissected and fixed (4% PFA for 30 minutes) following by washing (three times with wash buffer (0.02% NP-40, 0.01% deoxycholate in PBS) and chromogenic staining with staining solution (5 mM K3Fe(CN)6, 5 mM K4Fe(CN)6, 0.02% NP-40, 0.01% deoxycholate, 2 mM MgCl2, 5 mM EGTA, 1 mg/mL X-gal in PBS) in the dark at 37°C overnight.

### Gene transduction and transfection

For the generation of stable and constitutive cell lines with overexpression or knockdown of Cep55, we used Flag-*CEP55* cloned into the pLenti PGK Hygro Dest vector (addgene#19066), or mouse small-hairpin RNAs (shRNAs) in the pLKO plasmid (Sigma Aldrich, St Louis, USA). Cells were transduced by spinfection for 1 h in the presence of Hexadimethrine bromide (Polybrene) (Sigma Aldrich, St Louis, USA) and media collected and filtered at 48 h and 72 h post-transfection. For human cell lines, constitutive CEP55-knockdown was performed as previously described [1]. The Selection of clones was performed using 400 μg/mL Hygromycin, 50 μg/mL Zeocin, 5 μg/mL Blastocydin (Life Technologies) or 5 μg/mL of Puromycin (Life Technologies). Transient Cep55 silencing was performed by reverse transfection using 10–20 nM of individual small interfering RNAs (siRNAs manufactured by Shanghai Gene Pharma, China) and Lipofectamine RNAiMAX (Life Technologies) for 48 hours.

### Adenoviral shRNA infections

Cerebral brain organoids were infected with adenoviral shRNA viruses as per manufacturer’s instructions (Vector Biolabs). Two control and CEP55 adenoviral shRNAs were used: scrambled control Ad-U6-RNAi (cat# 1640), CMV driven Ad-GFP control (cat# 1060) and human CEP55 shRNA silencing adenovirus (cat# shADV-204994). Day 16 cerebral organoids were infected with the control and CEP55 adenoviral shRNAs at a MOI of 10. Organoids were harvested 24- and 48-hours post-infection to characterize knockdown of CEP55.

### Retrovirus and lentivirus packaging and transduction

For the production of retrovirus or lentivirus, Phoenix Amphotropic (retrovirus) or HEK293T cells (lentivirus) were plated at 90% confluency in a T75 flask and transfected with 5 μg of DNA and 15 μL Polyethylenimine or PEI (Polysciences, Inc., 23966–2, POL) (1:3 ratio) in Optimum media. At 5 h post-transfection, media was changed and the packaging cells incubated for 72 hours. At 48 hours and 72 hours post-transfection, the media was filtered using a 0.45 μm filter onto target cells prior to spinfection (at 1000 X g for 1 h at 25° C) in the presence of hexadimethrine bromide (polybrene; Sigma Aldrich, H9268-5G). Media was removed after spinfection and cells were allowed to recover for the next 48 h before selection with the corresponding antibiotic was carried out to select for transduced cells. Antibiotic selection was sustained until an untransduced control plate of cells had all died.

### Sequences for *Cep55* siRNA and ShRNA

(5’-3’) Cep55_Scr (CCGGCGCTGTTCTAATGACTAGCATCTCGAGATGCTAGTCATT AGAACAGCGTTTTTT); Cep55_sh#2 (CCGGCAGCGAGAGGCCTACGTTAAACTCG AGTTTAACGTAGGCCTCTCGCTGTTTTTG); Cep55_sh#4 (CCGGGAAGATTGAATC AGAAGGTTACTCGAGTAACCTTCTGATTCAATCTTCTTTTTT); SiRNA (Cep55_Scr Sense (5’-3’): CAAUGUUGAUUUGGUGUCUGCA) and anti-sense (5’-3’): UGAAU AGGAUUGUAAC); SiRNA Cep55_SEQ1 Sense (5’-3’): CCAUCACAGAGCAGCC AUUCCCACT and anti-sense (5’-3’): AGUGGGAAUGGCUGCUCUGUGAUG GUA)

### Cell and tissue lysate preparation

For preparation of tissue lysate, the organs were diced using a sterile scalpel blade followed by lysis in RIPA buffer (25mM Tris-HCl (pH 7.6), 150mM NaCl, 1% NP40, 1% Sodium deoxycholate and 0.1% SDS) or Urea lysis buffer (8M urea, 1% SDS, 100mM NaCl, 10mM Tris) and the samples were sonicated for 10 seconds on a Branson Sonifier 450 (Branson Ultrasonic Corporation, Danbury, CT, USA). Cell debris was removed by centrifugation at 13,000 RPM at 4°C for 30 minutes. Protein concentration was determined using a Pierce BCA Protein Assay Kit with Bio-Rad Protein Assay Dye Reagent (Thermo-Scientific). 30 or 60 μg of protein was resuspended in 1X laemmli buffer and samples heated to 95°C for 5 minutes prior to electrophoresis.

### Western blot

Western blotting was performed as previously described [3]. (Prepared protein samples were subjected to electrophoresis at 120 V using the Bio-Rad Mini-PROTEAN Tetra system in SDS running buffer (25 mM Tris-HCl, 192 mM glycine, 0.1% SDS (v/v)). Gel transfer was performed using the Invitrogen X-cell SureLock transfer system at 80 V for 90 minutes in 1X transfer buffer (50 mM Tris, 40 mM Glycine, 20% methanol) onto Amersham Hybond nitrocellulose membrane (GE Healthcare, Waukesha, WI, USA), and transfer efficiency assessed by Ponceau S staining (0.1% (w/v) Ponceau S in 5% acetic acid). Membranes were blocked in blocking buffer (5% Skim milk powder (Diploma Brand) in PBS containing 0.5% Tween-20- PBS-T) for 1 hour on a shaker at RT followed by overnight incubation with primary antibodies at 4° C. The membranes were washed (3x PBS-T) and incubated in secondary antibodies for 1 hour. Protein detection was performed using Super Signal chemiluminescent ECL-plus (PerkinElmer, Waltham, MA, USA) on a BioRad Gel doc (Bio-Rad ChemiDoc Touch, USA).

### Antibodies for Western blot

Cep55 (1:1000, In-house raised in rabbit against murine Cep55 (amino acids 55–250) and The Cell Signaling Technology, CEP55 (D1L4H) Rabbit mAb #81693 1:1000), Vinculin (1:2000; 13901 Cell Signaling Technology), β-Actin (1:2000, 612656 BD Pharmingen), β-Catenin (1:1000, 9582 Cell Signaling Technology), GSK 3β (1:1000, 9369 Cell Signaling Technology), pGSK 3β(Ser9) (1:1000, 9322 Cell Signaling Technology), Cleaved Caspase-3 (1:500, 9664 Cell Signaling Technology), pAKT(s473) (1:1000, 4060 Cell Signaling Technology), AKT (1:1000, 9271 Cell Signaling Technology), p Myc (T58) (1:1000, ab28842 Abcam), Non-phospho (Active) β-Catenin (Ser33/37/Thr41) (1:1000, 8814 Cell Signaling Technology), MYC (Y69) (1:1000, Ab32072Abcam).

### Statistical analysis

The sample sizes were determined based on previously published studies that performed similar analyses using samples sizes that are widely accepted [57] and demonstrated to be sufficient to reveal differences in cortical phenotypes in organoids [58] and in vivo experiments [48,59]. The sample size for in vivo embryonic analysis of developing neocortex was 4/ group which can detect differences of 2.38 standard deviations with 80% power. Two-tailed unpaired or paired Student’s t-test, one-way (Kruskal–Wallis test) or two-way ANOVA with post hoc Bonferroni, log-rank testing was performed as indicated using Prism v8.0 (Graph Pad Software, La Jolla, CA, USA) and the P-values were calculated as indicated in the figure legends. Mean and standard error of mean (Mean ± SD) are used to describe the variability within the sample in our analysis. ns; P > 0.05; *P ≤ 0.05; **P ≤ 0.01, ***P ≤ 0.001, and ****P ≤ 0.0001.

## Supporting information

S1 Fig Targeted disruption of *Cep55* gene in mice. **(A)** Schematic representation of murine *Cep55* gene loci: Wt (wild type, *Cep55*^+/+^), transgenic (gene trapped knockout first allele, *tm1a*,), showing the selection cassette (neo), the gene trapping cassette (LacZ), and LoxP and FRT recombination sites. The blue arrows indicate genotyping primers. The structure of Cep55 protein domains illustrates the coiled coil domain 1 (CC1), ESCRTs and ALIX-binding domain (EABR), the coiled coil domain 2 (CC2) and ubiquitin binding domain (UBD) **(B)** PCR genotyping showing *Cep55*^*+/+*^, *Cep55*^*+/-*^ and *Cep55*^*-/-*^ genotypes. **(C)** mRNA expression of *Cep55* in *Cep55*^*+/+*^and *Cep55*^*-/-*^ E14.5 mouse heads for indicated primers binding *Cep55’*s exon 3–4 (left) and exon 6–7 (right). *ACTB* was used as a housekeeping gene for normalization. Data represent the mean ± SD, n = 2 mice per genotype, 3 independent experiments, Student’s t-tes, p< 0.1404 (left) and p< <0.0001 (right); *P < 0.05, **P < 0.01, ***P < 0.001, ****P < 0.0001). **(D)** Immunoblot analysis of Cep55 protein expression from *Cep55*^*+/+*^, *Cep55*^*+/-*^ and *Cep55*^*-/-*^ E14.5 mouse heads. β-actin was used as a loading control. **(E)** Comparison of organ volumes of 8-week-old *Cep55*^+/+^ and *Cep55*^+/-^ mice. Brain and thymus size are slightly smaller in *Cep55*^+/-^ (Het) mice, n = 2 per group. **(F)** Mean body weights of *Cep55*^+/+^ and *Cep55*^+/-^ offspring measured at the indicated time points until 20 weeks. n = 6–9 mice per group. **(G)** *Cep55* expression in the single-cell transcriptomic analysis of mouse neocortical development visualized based on the available data at Zylka lab dataset. The highest expression is seen in radial-glial cells (RG2) at embryonic day 14. **(H)** β-galactosidase staining of coronal sections of *Cep55*+/-mouse embryonic brain at the indicated time points. Dotted black box indicates the magnified area shown on right, Scale = 100 μm.
(TIF)

S2 Fig The phenotypic analysis of *Cep55^-/-^* mouse brain and human organoid. **(A)** Cerebellar hypoplasia in *Cep55*^-/-^ (lower panel) compared to *Cep55*^+/+^ (upper panel) brain sections. Compared to the *Cep55*^+/+^, there is a marked reduction in thickness of the external granular layer (EGL) in a *Cep55*^-/-^ brain The cerebellar cortical neuronal populations are deficient and disorganized in *Cep55*^-/-^ neocortex. The higher power views (of the boxed area) show the thickness of EGL in the cerebellar cortices of *Cep55*^-/-^ compared to *Cep55*^+/+^ mice (right). The olfactory bulb is also neuron-deficient and disorganized in a *Cep55*^-/-^ mouse compared to a *Cep55*^+/+^ mouse (left). Scale = 60μm. **(B)** Comparison of cerebral hemisphere (neocortex (NCx), germinal epithelium (GE) and lateral ventricles) from Cep55^+/+^ (upper) and Cep55^-/-^ (lower) E18.5 embryos. Red arrows indicate structural dilation, distortion and disorganization, and necrotic area with neural tissue loss, scale = 200μm. Middle: magnification of boxed area showing depletion of subependymal germinal neuroblasts in Cep55^-/-^, scale = 50μm. Right: magnification of boxed area showing neocortical neuronal depletion in cerebral hemispheres and reduction of cortical neuronal population in Cep55^-/-^. Red arrow identifies multinucleated neurons. Scale = 20μm. **(C)** Hematoxylin and eosin staining of E18.5 Cep55^-/-^ cerebral cortex. Upper: neocortical hypoplasia/dysplasia. Diminished and disorganized neurons with an area of parenchymal necrosis (N, black arrow) and neural tissue loss. Phagocytosed neuronal cellular debris is arrowed and magnified. Scale = 120μm. Lower: numerous bi-nucleated neurons (red arrows), scale = 180μm. **(D)** Representative image of NeuN (brown) and Eosin (pink) immunohistochemical staining of E18.5 sections from *Cep55*^+/+^ (left) and *Cep55*^-/-^ (right) E18.5 embryonic brain sections showing multinucleation, scale = 50μm. **(E)** Graphical representation of percentage of total cells showing multinucleation. **(F)** Immunoblotting showing cleaved caspase 3 expression in *Cep55*^+/+^ and *Cep55*^-/-^ MEFs. β actin was used as a loading control. **(G)** The enlarged macro images of iPSC-derived human cerebral brain organoids, note the neuroepithelial structures observed in differentiated brain organoid, scale = 100μm. **(H)** Immunofluorescence staining of organoid section showing neuroprogenitor markers: PAX6 (magenta, TBR2 (Green) and DAPI (blue), in the left panel and CTIP-1 (magenta), TBR1 (yellow) and DAPI (blue), in the right panel, scale = 20μm. **(I)** Immunofluorescence staining of cilia in cerebral brain organoids infected with U6 scrambled control (left), and adenoviral shRNAs *CEP55* knockdown (KD) (right), ARL13b (green),γ tubulin (red), DAPI (blue), scale = 15μm.
(TIF)

S3 Fig Defective ciliogenesis in *Cep55^-/-^* mouse neocortex (E14.5) and MEFs. **(A)** Representative image of E14.5 mouse neocortex immunostained for cilia (Arl13b), basal body (Ɣ-tubulin), Cep55 and DAPI from *Cep55*^+/+^ (upper) and *Cep55*^-/-^ (lower) mice, each channel and merged image are shown, notice that Cep55 signals could not be detected in Cep55^-/-^ (left), Bar chart shows cilia-positive cells in Ncx in the 100 μm-width box at E14.5, cilia counts normalized to total cell (DAPI) number (lower) at E14.5 (right). **(B)** Cilia-positive cells in Ncx in the 100 μm-width box at E18.5, quantification of ciliated IPCs (left) and RGCs (right) in the neocortex, expressed as a ratio of total cell numbers. Cell numbers were obtained from data shown in Figs S3A and 2A and 2B (Mean ± SD of four embryos measured in duplicate, Student’s t-test, *P < 0.05, **P < 0.01, ***P < 0.001, ****P < 0.0001). **(C)** Representative images of RPE-1 cells transiently transfected with si-Scramble (left panel) or siRNA against *CEP55* for 48 h (right panel) showing cilia (ARL13b) and nuclei (DAPI). Bar chart shows a comparison of percentage of ciliated cells. **(D)** Immunoblot of *CEP55* expression in Ctrl (Empty vector), or CEP55-depleted (shRNA CEP55) RPE-1 cells. Vinculin was used as a loading control. **(E)** Immunoblot of *Cep55* expression in *Cep55*^+/+^ and *Cep55*^*-*/-^ MEFs, Vinculin was used as a loading control, **(F)** Representative images of different phenotypes of cilia in *Cep55*^+/+^ and *Cep55*^*-*/-^ MEFs (shortened cilia, double cilia and remnant cilia). Bar charts show percentage ciliated cells, cilia number and percent of cells with remnant cilia or double cilia. (Mean ± SD, n = 300 cilia per group of 2 independent experiments. Student’s t-test, *P < 0.05, **P < 0.01, ***P < 0.001, ****P < 0.0001), scale = 10μm. **(G)** Immunoblotting showing *Cep55* expression in *Cep55*^+/+^, *Cep55*^-/-^ MEFs without or with reconstituted CEP55 (rescue). Vinculin was used as a loading control. **(H)** Representative images of individual channels showing cilia (Arl13b, green), basal body (Ɣ-tubulin,red), DAPI (blue) as well as cilia (green) and Cep55 (yellow) and DAPI (blue) showing the co-localization of Cep55 and Ɣ-tubulin at the base of cilia. Scale = 5 μm.
(TIF)

S4 Fig Analysis of proliferation, mitosis and cell cycle defects in *Cep55^-/-^* MEFs. **(A)** Doubling time of *Cep55*^+/+^ and *Cep55*^*-*/-^ MEFs (Mean ± SD, n = 2 biological repeats and 3 independent experiments Student’s t-test, ****P < 0.0001). **(B-C)** Proliferation of **(B)** *Cep55*^+/+^, *Cep55*^+/-^ and *Cep55*^*-*/-^ MEFs and **(C)** *Cep55*^+/+^ (Wt), *Cep55*^*-*/-^ (KO) and *CEP55*-reconstituted (Rescue) MEFs (Mean ± SEM, average of 2 biological repeats and 2 independent experiments Student’s t-test, ****P < 0.0001), measured using IncuCyte, Corresponding immunoblotting for *Cep55* expression is shown below each graph. Vinculin was used as a loading control. **(D)** Representative images of individual channels showing α-tubulin (cytoskeleton), Cep55, and nuclei (DAPI) in *Cep55*^+/+^ (left) and *Cep55*^*-/-*^ (right) MEFs. **(E)** Bar chart showing percent of multinucleated cells in constitutive MEF (*Cep55*^+/+^ (wt), *Cep55*^+/-^ (Het) and *Cep55*^-/-^(KO)), (Mean ± SD, n = 300 cells counted from 2 biological repeats and 3 independent experiments, One-Way ANOVA test, *P < 0.05, **P < 0.01, ***P < 0.001). **(F)** Modfit histogram of cell cycle analysis by FACS showing cell cycle distribution of *Cep55*^+/+^ (left) and *Cep55*^-/-^ (right) MEFs. **(G)** Graph showing percent of cells in G1, S and G2 for each genotype. Data represent mean ± SD of two lines per genotype, measured in duplicate across three independent experiments. **(H)** Representative images from time-lapse microscopy of *Cep55*^+/+^ (upper panel) and *Cep55*^-/-^ (lower panel) MEFs transfected with mCherry-histone H2B showing different phases of mitosis and cytokinesis. **(I)** Dot plot showing the time cells take to complete the mitosis (left), the stacked bar chart showing the average time to complete cell division (right) for *Cep55*^+/+^ and *Cep55*^-/-^ MEFs. **(J)** Column chart showing the percentage of cells with cytokinesis failure (multinucleated cells) or success (single cells) for *Cep55*^+/+^ and *Cep55*^-/-^, (Mean ± SD, n = 10–25 cells counted from 3 technical repeats Student’s t-test, **P < 0.01). **(K)** The stacked bar chart represents a comparison of percentages of different mitotic phenotypes of MEFs transfected with Cherry-histone H2B for *Cep55*^+/+^ (left) and *Cep55*^-/-^ (right), based on images of the cell captured by time-lapse microscopy (Spinning disk confocal microscopy). (Mean ± SD, n = 55–67 cells counted from 3 technical repeats Student’s t-test, **P < 0.01).
(TIF)

S5 Fig Reduced Myc transcript levels in *Cep55^-/-^* mouse brain (E14.5) / MEFs and the analysis of the impact of GSK3β inhibition on proliferation and ciliogenesis. **(A-B)** Fold change of mRNA expression of the indicated transcripts for **(A)** *Cep55*^+/+^ and *Cep55*^-/-^ E14.5 brain extracts and **(B)** MEFs. **(C)** Immunoblot showing expression of Akt in EV and myrAKT transfected Cep55^-/-^ MEF. Vinculin was used as a loading control. Proliferation assay showing growth of **(D)** *Cep55*^+/+^ (Wt, left), and Flag-*CEP55* reconstituted *Cep55*^-/-^ MEFs (Rescue, right) treated with indicated doses of GSK3β inhibitor, CHIR99021 (untreated: red, 0.1 μM inhibitor: green, 1 μM inhibitor: blue), (Mean ± SD, average of 2 biological repeats and 2 independent experiments Student’s t-test, ****P < 0.0001). **(E)** Representative images of *Cep55*^+/+^(left) and *Cep55*^-/-^ (right) MEFs untreated (upper) and treated (lower) with 1μM of GSK3β inhibitor, CHIR99021. Bar chart shows the percentage of ciliated cells in *Cep55*^+/+^ and *Cep55*^-/-^ MEFs untreated and treated with 1μM of GSK3β inhibitor, CHIR99021, n = 100.
(TIF)

S1 Table Proportion of observed and expected offspring from *Cep55^+/-^* x *Cep55^+/-^* intercrosses. (DOCX)

S2 Table Number and percentage of offspring at indicated stages of gestation from *Cep55^+/-^* X *Cep55^+/-^* intercrosses. (DOCX)
