# Supplementary figures and images for: Cep55 regulation of PI3K/Akt signaling is required for neocortical development and ciliogenesis
Source: PLoS Genet. 2021 Oct 28;17(10):e1009334. doi: 10.1371/journal.pgen.1009334 (PMC8577787; doi:10.1371/journal.pgen.1009334)

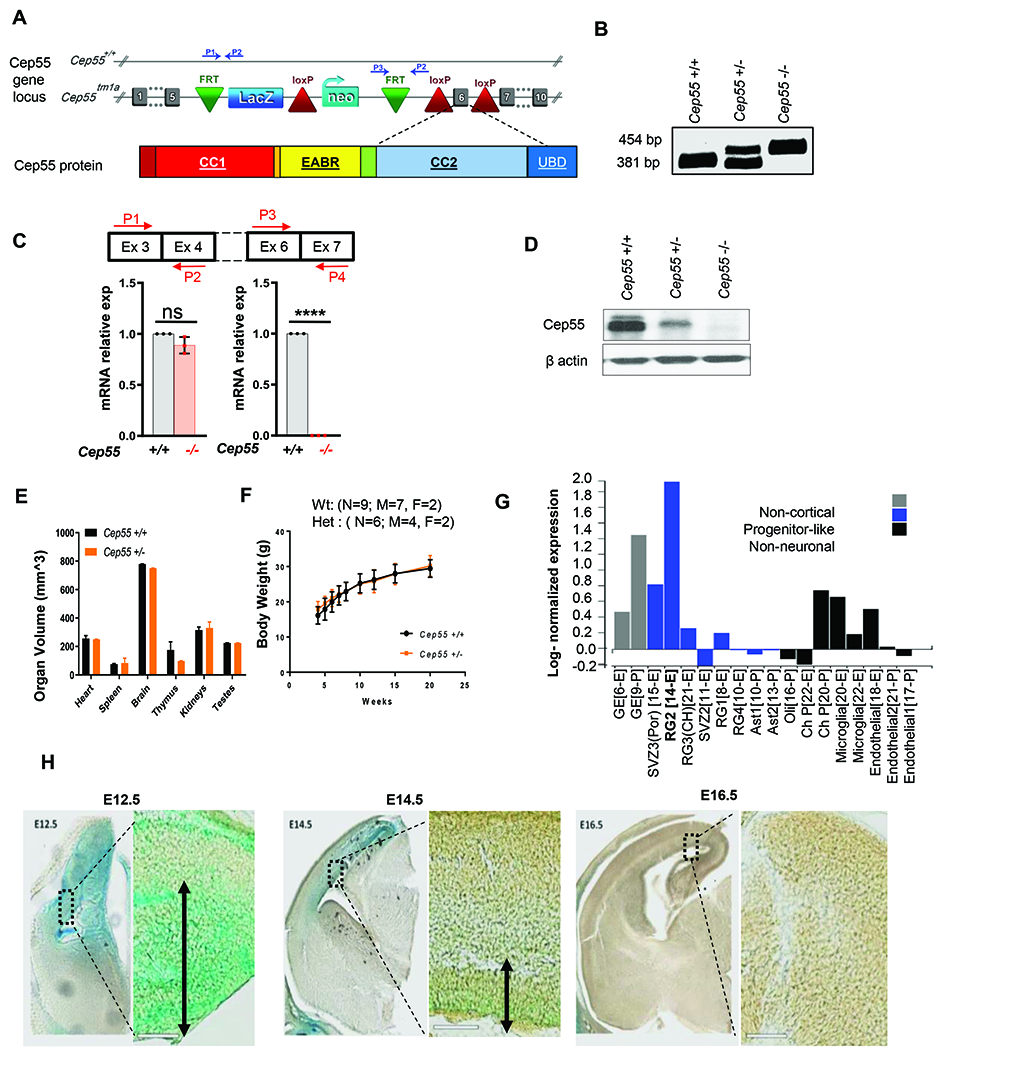

Supplement: S1 Fig — (A) Schematic representation of murine Cep55 gene loci: Wt (wild type, Cep55+/+), transgenic (gene trapped knockout first allele, tm1a,), showing the selection cassette (neo), the gene trapping cassette (LacZ), and LoxP and FRT recombination sites. The blue arrows indicate genotyping primers. The structure of Cep55 protein domains illustrates the coiled coil domain 1 (CC1), ESCRTs and ALIX-binding domain (EABR), the coiled coil domain 2 (CC2) and ubiquitin binding domain (UBD) (B) PCR genotyping showing Cep55+/+, Cep55+/- and Cep55-/- genotypes. (C) mRNA expression of Cep55 in Cep55+/+and Cep55-/- E14.5 mouse heads for indicated primers binding Cep55’s exon 3–4 (left) and exon 6–7 (right). ACTB was used as a housekeeping gene for normalization. Data represent the mean ± SD, n = 2 mice per genotype, 3 independent experiments, Student’s t-tes, p< 0.1404 (left) and p< <0.0001 (right); *P < 0.05, **P < 0.01, ***P < 0.001, ****P < 0.0001). (D) Immunoblot analysis of Cep55 protein expression from Cep55+/+, Cep55+/- and Cep55-/- E14.5 mouse heads. β-actin was used as a loading control. (E) Comparison of organ volumes of 8-week-old Cep55+/+ and Cep55+/- mice. Brain and thymus size are slightly smaller in Cep55+/- (Het) mice, n = 2 per group. (F) Mean body weights of Cep55+/+ and Cep55+/- offspring measured at the indicated time points until 20 weeks. n = 6–9 mice per group. (G) Cep55 expression in the single-cell transcriptomic analysis of mouse neocortical development visualized based on the available data at Zylka lab dataset. The highest expression is seen in radial-glial cells (RG2) at embryonic day 14. (H) β-galactosidase staining of coronal sections of Cep55+/-mouse embryonic brain at the indicated time points. Dotted black box indicates the magnified area shown on right, Scale = 100 μm. (TIF) [file pgen.1009334.s001.tif]

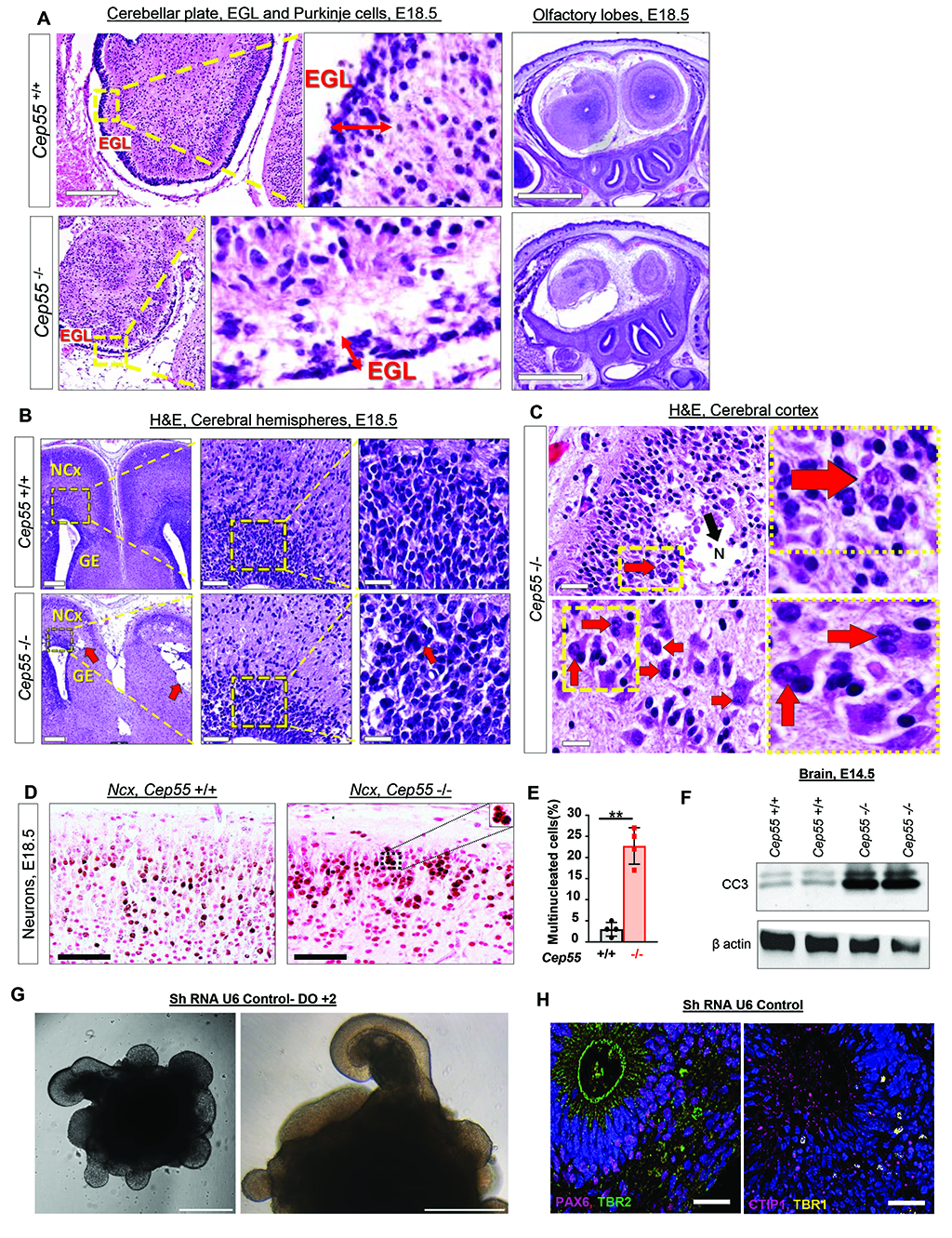

Supplement: S2 Fig — (A) Cerebellar hypoplasia in Cep55-/- (lower panel) compared to Cep55+/+ (upper panel) brain sections. Compared to the Cep55+/+, there is a marked reduction in thickness of the external granular layer (EGL) in a Cep55-/- brain The cerebellar cortical neuronal populations are deficient and disorganized in Cep55-/- neocortex. The higher power views (of the boxed area) show the thickness of EGL in the cerebellar cortices of Cep55-/- compared to Cep55+/+ mice (right). The olfactory bulb is also neuron-deficient and disorganized in a Cep55-/- mouse compared to a Cep55+/+ mouse (left). Scale = 60μm. (B) Comparison of cerebral hemisphere (neocortex (NCx), germinal epithelium (GE) and lateral ventricles) from Cep55+/+ (upper) and Cep55-/- (lower) E18.5 embryos. Red arrows indicate structural dilation, distortion and disorganization, and necrotic area with neural tissue loss, scale = 200μm. Middle: magnification of boxed area showing depletion of subependymal germinal neuroblasts in Cep55-/-, scale = 50μm. Right: magnification of boxed area showing neocortical neuronal depletion in cerebral hemispheres and reduction of cortical neuronal population in Cep55-/-. Red arrow identifies multinucleated neurons. Scale = 20μm. (C) Hematoxylin and eosin staining of E18.5 Cep55-/- cerebral cortex. Upper: neocortical hypoplasia/dysplasia. Diminished and disorganized neurons with an area of parenchymal necrosis (N, black arrow) and neural tissue loss. Phagocytosed neuronal cellular debris is arrowed and magnified. Scale = 120μm. Lower: numerous bi-nucleated neurons (red arrows), scale = 180μm. (D) Representative image of NeuN (brown) and Eosin (pink) immunohistochemical staining of E18.5 sections from Cep55+/+ (left) and Cep55-/- (right) E18.5 embryonic brain sections showing multinucleation, scale = 50μm. (E) Graphical representation of percentage of total cells showing multinucleation. (F) Immunoblotting showing cleaved caspase 3 expression in Cep55+/+ and Cep55-/- MEFs. β actin was us [file pgen.1009334.s002.tif]

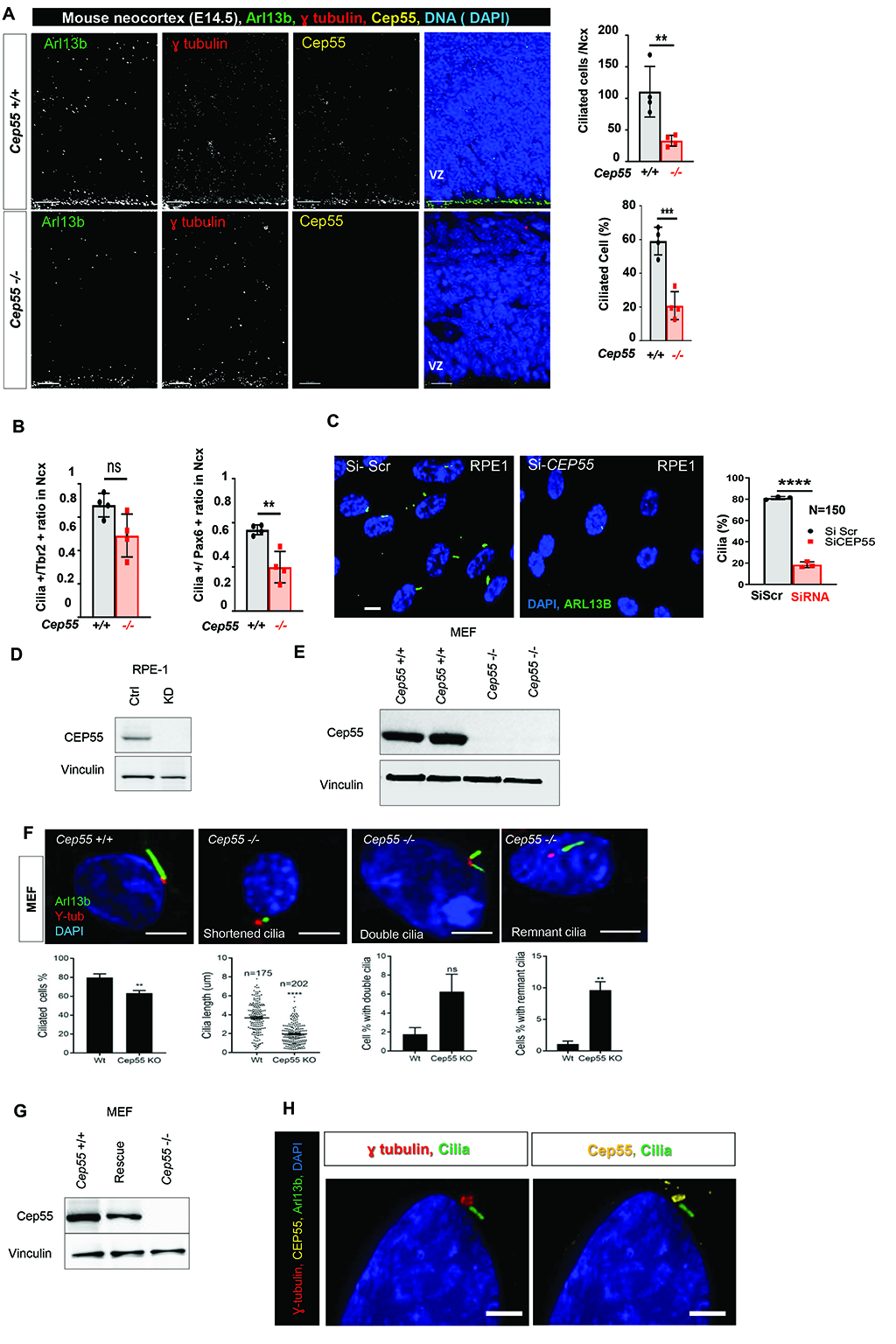

Supplement: S3 Fig — (A) Representative image of E14.5 mouse neocortex immunostained for cilia (Arl13b), basal body (Ɣ-tubulin), Cep55 and DAPI from Cep55+/+ (upper) and Cep55-/- (lower) mice, each channel and merged image are shown, notice that Cep55 signals could not be detected in Cep55-/- (left), Bar chart shows cilia-positive cells in Ncx in the 100 μm-width box at E14.5, cilia counts normalized to total cell (DAPI) number (lower) at E14.5 (right). (B) Cilia-positive cells in Ncx in the 100 μm-width box at E18.5, quantification of ciliated IPCs (left) and RGCs (right) in the neocortex, expressed as a ratio of total cell numbers. Cell numbers were obtained from data shown in Figs S3A and 2A and 2B (Mean ± SD of four embryos measured in duplicate, Student’s t-test, *P < 0.05, **P < 0.01, ***P < 0.001, ****P < 0.0001). (C) Representative images of RPE-1 cells transiently transfected with si-Scramble (left panel) or siRNA against CEP55 for 48 h (right panel) showing cilia (ARL13b) and nuclei (DAPI). Bar chart shows a comparison of percentage of ciliated cells. (D) Immunoblot of CEP55 expression in Ctrl (Empty vector), or CEP55-depleted (shRNA CEP55) RPE-1 cells. Vinculin was used as a loading control. (E) Immunoblot of Cep55 expression in Cep55+/+ and Cep55-/- MEFs, Vinculin was used as a loading control, (F) Representative images of different phenotypes of cilia in Cep55+/+ and Cep55-/- MEFs (shortened cilia, double cilia and remnant cilia). Bar charts show percentage ciliated cells, cilia number and percent of cells with remnant cilia or double cilia. (Mean ± SD, n = 300 cilia per group of 2 independent experiments. Student’s t-test, *P < 0.05, **P < 0.01, ***P < 0.001, ****P < 0.0001), scale = 10μm. (G) Immunoblotting showing Cep55 expression in Cep55+/+, Cep55-/- MEFs without or with reconstituted CEP55 (rescue). Vinculin was used as a loading control. (H) Representative images of individual channels showing cilia (Arl13b, green), basal body (Ɣ-tubulin,red), DAPI (blue) as well as [file pgen.1009334.s003.tif]

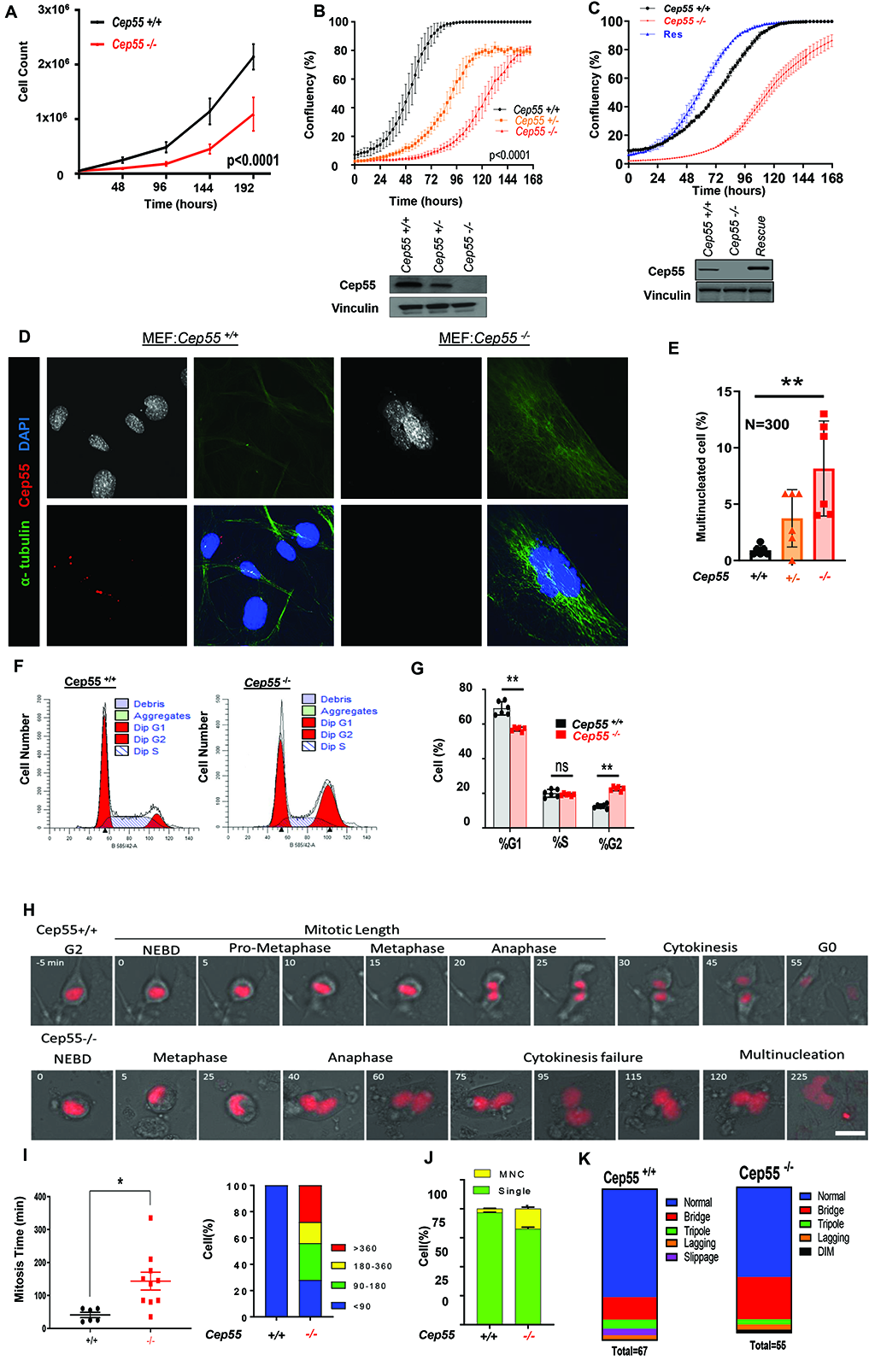

Supplement: S4 Fig — (A) Doubling time of Cep55+/+ and Cep55-/- MEFs (Mean ± SD, n = 2 biological repeats and 3 independent experiments Student’s t-test, ****P < 0.0001). (B-C) Proliferation of (B) Cep55+/+, Cep55+/- and Cep55-/- MEFs and (C) Cep55+/+ (Wt), Cep55-/- (KO) and CEP55-reconstituted (Rescue) MEFs (Mean ± SEM, average of 2 biological repeats and 2 independent experiments Student’s t-test, ****P < 0.0001), measured using IncuCyte, Corresponding immunoblotting for Cep55 expression is shown below each graph. Vinculin was used as a loading control. (D) Representative images of individual channels showing α-tubulin (cytoskeleton), Cep55, and nuclei (DAPI) in Cep55+/+ (left) and Cep55-/- (right) MEFs. (E) Bar chart showing percent of multinucleated cells in constitutive MEF (Cep55+/+ (wt), Cep55+/- (Het) and Cep55-/-(KO)), (Mean ± SD, n = 300 cells counted from 2 biological repeats and 3 independent experiments, One-Way ANOVA test, *P < 0.05, **P < 0.01, ***P < 0.001). (F) Modfit histogram of cell cycle analysis by FACS showing cell cycle distribution of Cep55+/+ (left) and Cep55-/- (right) MEFs. (G) Graph showing percent of cells in G1, S and G2 for each genotype. Data represent mean ± SD of two lines per genotype, measured in duplicate across three independent experiments. (H) Representative images from time-lapse microscopy of Cep55+/+ (upper panel) and Cep55-/- (lower panel) MEFs transfected with mCherry-histone H2B showing different phases of mitosis and cytokinesis. (I) Dot plot showing the time cells take to complete the mitosis (left), the stacked bar chart showing the average time to complete cell division (right) for Cep55+/+ and Cep55-/- MEFs. (J) Column chart showing the percentage of cells with cytokinesis failure (multinucleated cells) or success (single cells) for Cep55+/+ and Cep55-/-, (Mean ± SD, n = 10–25 cells counted from 3 technical repeats Student’s t-test, **P < 0.01). (K) The stacked bar chart represents a comparison of percentages of different mitotic pheno [file pgen.1009334.s004.tif]

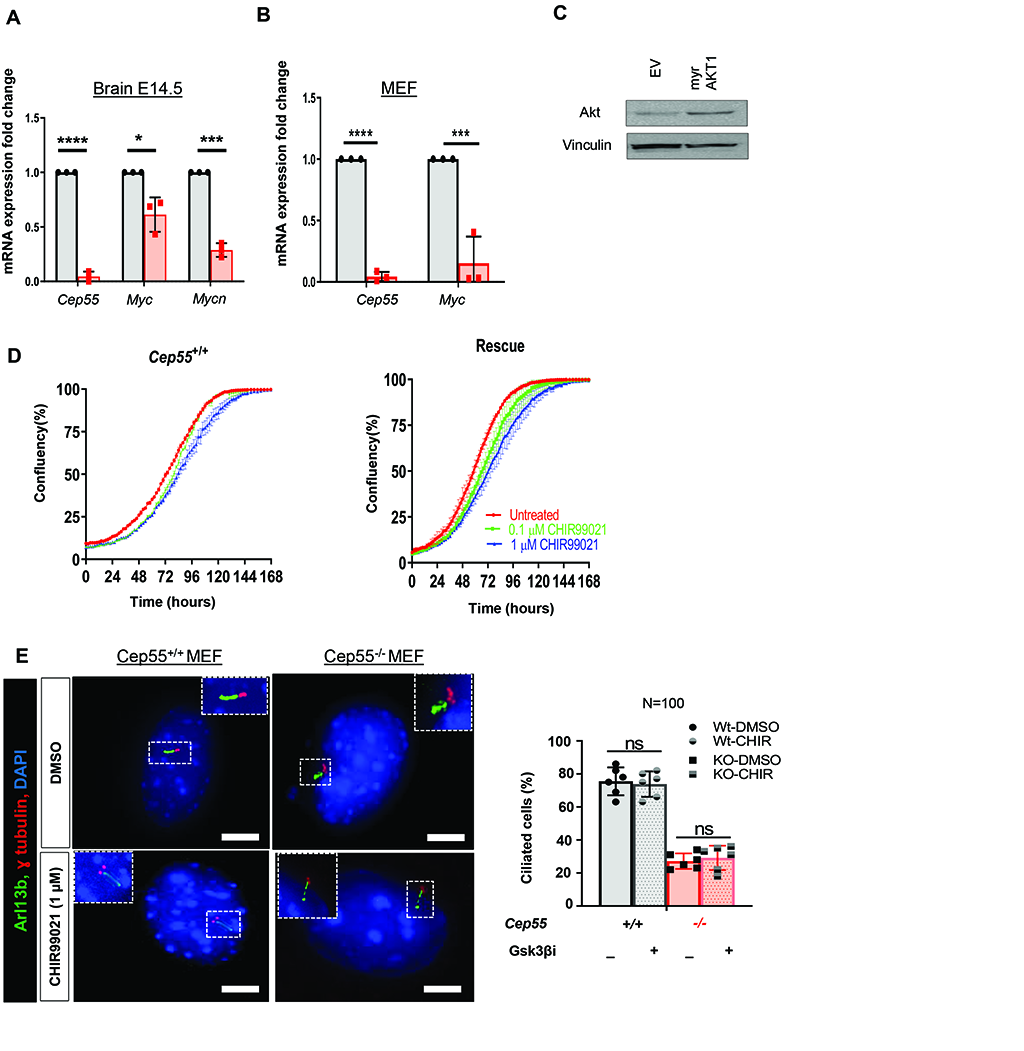

Supplement: S5 Fig — (A-B) Fold change of mRNA expression of the indicated transcripts for (A) Cep55+/+ and Cep55-/- E14.5 brain extracts and (B) MEFs. (C) Immunoblot showing expression of Akt in EV and myrAKT transfected Cep55-/- MEF. Vinculin was used as a loading control. Proliferation assay showing growth of (D) Cep55+/+ (Wt, left), and Flag-CEP55 reconstituted Cep55-/- MEFs (Rescue, right) treated with indicated doses of GSK3β inhibitor, CHIR99021 (untreated: red, 0.1 μM inhibitor: green, 1 μM inhibitor: blue), (Mean ± SD, average of 2 biological repeats and 2 independent experiments Student’s t-test, ****P < 0.0001). (E) Representative images of Cep55+/+(left) and Cep55-/- (right) MEFs untreated (upper) and treated (lower) with 1μM of GSK3β inhibitor, CHIR99021. Bar chart shows the percentage of ciliated cells in Cep55+/+ and Cep55-/- MEFs untreated and treated with 1μM of GSK3β inhibitor, CHIR99021, n = 100. (TIF) [file pgen.1009334.s005.tif]
