# Supplementary material for: Cep55 regulation of PI3K/Akt signaling is required for neocortical development and ciliogenesis
Source: PLoS Genet. 2021 Oct 28;17(10):e1009334. doi: 10.1371/journal.pgen.1009334 (PMC8577787; doi:10.1371/journal.pgen.1009334)
Supplement: S1 Table — (DOCX) [file pgen.1009334.s006.docx]

| **Postweaning** | ***Cep55*^+/+^** | ***Cep55*^+/-^** | ***Cep55*^-/-^** | **Total** |
| --- | --- | --- | --- | --- |
| **Observed** | 138 | 183 | 0 | 321 |
| **%** | 43.0% | 57.0% | 0% | 100% |
| **Expected** | 80.25 | 160.5 | 80.25 | 321 |
| **%** | 25% | 50% | 25% | 100% |

**S1 Table.** Proportion of observed and expected offspring from *Cep55^+/-^* x *Cep55^+/-^* intercrosses.
