# Supplementary material for: Cep55 regulation of PI3K/Akt signaling is required for neocortical development and ciliogenesis
Source: PLoS Genet. 2021 Oct 28;17(10):e1009334. doi: 10.1371/journal.pgen.1009334 (PMC8577787; doi:10.1371/journal.pgen.1009334)
Supplement: S2 Table — (DOCX) [file pgen.1009334.s007.docx]

| **Embryonic Day/**  **Attempts** | | ***Cep55*^+/+^** | ***Cep55*^+/-^** | ***Cep55*^-/-^** | **Total** |
| --- | --- | --- | --- | --- | --- |
| **E11.5** | 1 | 6 | 1 | 3 | 10 |
| % | | 60% | 10% | 30% | 100% |
| **E13.5** | 7 | 18 | 20 | 16 | 54 |
| % | | 33.3% | 37% | 29.6% | 100% |
| **E14.5** | 4 | 5 | 17 | 6 | 28 |
| % | | 17.9% | 60.7% | 21.4% | 100% |
| **E15.5** | 1 | 3 | 5 | 3 | 11 |
| % | | 27.3% | 45.5% | 27.3% | 100% |
| **E16.5** | 3 | 7 | 14 | 4 | 25 |
| % | | 28% | 56% | 16% | 100% |
| **E18.5** | 4 | 11 | 14 | 12 | 37 |
| % | | 29.7% | 37.8% | 32.4% | 100% |
| **P0** | 4 | 12 | 16 | 1 | 29 |
| % | | 41.3% | 55.1% | 3.4% | 100% |

**S2 Table.** Number and percentage of offspring at indicated stages of gestation from *Cep55^+/-^* X *Cep55^+/-^* intercrosses.
